# Minimizing total costs for distribution power grids considering harmonics and uncertainty of wind speed and solar radiation

**DOI:** 10.1371/journal.pone.0355399

**Published:** 2026-09-11

**Authors:** Le Chi Kien, Nguyen Dinh Phu, Thang Trung Nguyen, Thai Dinh Pham, Minh Phuc Duong

**Affiliations:** 1 Faculty of Electrical and Electronics Engineering, Ho Chi Minh City University of Technology and Engineering, Ho Chi Minh City, Vietnam; 2 Power System Optimization Research Group, Faculty of Electrical and Electronics Engineering, Ton Duc Thang University, Ho Chi Minh City, Vietnam; 3 Faculty of Electrical and Electronics Engineering, Ho Chi Minh City University of Industry and Trade, Ho Chi Minh City, Vietnam; Huazhong University of Science and Technology, CHINA

## Abstract

Nowadays, distributed renewable energy generation sources are increasingly popular and strongly impact the traditional power grid. Therefore, determining the appropriate penetration of these sources is a big challenge because of the randomness of the renewable electricity output. This study applies a robust and stable algorithm, called the modified coyote optimization algorithm (MCOA), to determine an optimal solution for the effective integration of wind turbine and photovoltaic distributed generation units (WT-PVDGUs) in different-scale power grids of IEEE 33 buses and 69 buses. For the first case, the primary goal is to minimize the combined costs of investment, operation, and maintenance (OM) of WT-PVDGUs, energy purchase for loads, and energy loss during distribution while mitigating the harmfulness of harmonics on power quality and load operation. The study applies Weibull and Beta probability distribution functions (pdfs) to predict the output power of WT-PVDGUs to enhance the quality of the found feasible solutions. The obtained results from the suggested method are compared with five other active methods to demonstrate the superiority of MCOA with total cost savings of up to 25.56% ($4.1811 million) for the first system and 27.05% ($4.9090 million) for the second system over the 20-year project life cycle. In addition, in the second case, the optimal solution from MCOA achieves a loss reduction of up to 65.51% in the first system and 69.17% in the second system. These results are better than dozens of recently published methods. Not only that, but the voltage profile is also raised to the best range, and the total and individual harmonic distortions are mitigated and meet the IEEE Std. 519 thanks to the optimal installation of units in the power grids. Therefore, this study has significantly contributed to reducing total costs, eliminating the harmful effects of harmonics, and dealing with uncertainty regarding renewable energies.

## 1. Introduction

The gradual transition from fossil fuel energy to renewable energy is a major global challenge. The potential benefits from the use of renewable energy are enormous and promising for solving the problems that the world is facing, related to environmental protection and energy stability. Therefore, many countries around the world have policies to support the penetration of this energy source to increase the proportion of national energy generation [1]. Like Sweden, renewable energy penetration has increased steadily from 33% to 58% of total generation for the period 1990–2019, thanks to appropriate policies (In 2019, the country’s total electricity output amounted to 164.4 TWh), and the Swedish government also set a national target for reaching 100% renewable energy by 2040 [2]. Another country whose government is paying great attention to the use of renewable energy and reducing emissions is Japan. Japan is currently the fifth-largest emitter of greenhouse gases in the world, and the government has committed to net-zero emissions by 2050. To achieve this commitment, Japan has adapted strategies to suit the country’s specific circumstances for the purpose of increasing the electricity production from renewable energy to the range (50% − 60%) by 2050, 10% for Hydropower and (30% − 40%) for nuclear and fossil energies with the application of carbon capture and storage technology [3]. Moreover, larger countries like India and China have also significantly boosted the penetration of renewable energy. For example, the study [4] has stated that, in India, renewable energy production accounted for 28.04% in 2022, corresponding to 113.226 GW. Not only that, this country also aims to achieve 54% and 80% for the market share from renewable energy by 2030 and 2040, respectively. In addition, in China, carbon-neutral scenarios have also been proposed to reduce emissions and maximize renewables. The results from the research [5] show that this country has the potential to reach 60% of total energy consumption from renewables and 90% of total national generation by 2050. With the strong energy transition, air quality will improve with an 85% reduction in 2050 compared to 2020. Realistically, the penetration of renewable energy sources brings both benefits and challenges [6]. The use of renewables has many advantages in terms of reduced emissions, unlimited supply, and lower operating and maintenance costs. However, due to the uncertain nature of these sources in terms of dependence on environmental conditions, this leads to unstable power output, and it is difficult to predict generation for planning electricity distribution. Different popular approaches, such as mixed-integer linear programming (MILP), mixed-integer nonlinear programming (MINLP), and meta-heuristic methods (MHM), are also applied to determine the optimal penetration for maximizing the received benefits and limiting the negative impacts from connecting renewables into the power grid [7]. Researchers around the world have proved that integrating renewable sources as distributed generation units (DGUs) into the distribution systems (DSs) can be considered as one of the great solutions that bring effective economic-technical-environmental benefits [8,9]. However, connecting PVDGUs to DSs also causes many adverse effects due to changing the power grid structure, and the extent of this influence largely depends on the access location and installation power of DGUs. Thus, it is essential to determine the optimal the placement and efficiency of PVDGU in DSs [10].

Among the MILP, MINLP, and MHM groups, the MHM group offers many advantages in terms of performance, stability, and implementation structure over the remaining groups. Therefore, the MHM group is widely used for various optimization problems, especially the problem of finding an optimal solution for the integration of DGUs in the distribution network. A fuzzy classification algorithm based on RAO-3 was proposed for optimizing both DGUs and Shunt Capacitors after network reconfiguration [11]. PSO variants have been applied to determine the penetration of DGUs in the power grid to minimize branch losses and enhance voltage profiles [12]. The results of that research showed clear benefits from properly integrating DGUs into DSs. Although PSO is a popular algorithm, it has many disadvantages, such as low efficiency and no convergence guarantee, so several researchers have implemented effective changes through hybridization between the original method and another method to enhance its performance. Hybrid methods, such as analytical approach/PSO [13], PSO/GSA [14], and Fuzzy/PSO [15], have been successfully developed to tackle the installation problem of DGUs in the power grids. The simulation results demonstrated that the hybrid approach is superior to the original method. Similarly, the authors [16] also suggested using ABC to handle optimization problems. Those authors have considered the connection of DGUs into two DSs of 14 buses and 57 buses to analyze the change in loss reduction and voltage improvement. The results demonstrated that the amount of loss reduction and voltage enhancement depends on the number of DGUs that are connected to the grid. With the same applied method, other authors have focused on the economic aspect of successful minimization of the total energy cost, thanks to the appropriate determination in installing DGUs [17,18]. ABC is also a traditional and long-standing method, but its biggest drawback is that it is easy to fall into the local zone, like GA and PSO. Besides, due to the complex structure of this algorithm, when it has to go through all three bee phases, the data processing time for a feasible solution is longer than that of other methods. To overcome the disadvantages of ABC, an improved method called CABC was born. CABC is also applied to the considered optimization problem, and it successfully solved the voltage stability improvement problem as the main target with the high penetration level of DGUs in the power grid [19]. In another aspect of considering harmonic distortions from nonlinear loads, authors in [20,21] have also positively mitigated harmonics to the allowed limits in IEEE Std. 519 and enhanced the power quality by identifying the appropriate placement and capacity of DGUs. Additionally, more effective algorithms such as SSA [22] and GWO [23] have shown superior performance and stability in tackling real-world optimization problems with the high penetration of capacitors (CBs) for the purpose of reducing losses and improving voltage stability indices in distribution systems. Not only that, some other interesting studies have also addressed the problem of DGUs connection into smart microgrids. Solving the optimization problem for this type of grid is very complex [24–27]. The authors in [24] applied traditional algorithm (GA) combined with optimal tuning parameters to find the best solution for solar power systems and electric vehicle charging stations (EVCS) to minimize losses and increase the proximity of EVCS to the load center in the microgrid of a university in Brazil. Meanwhile, other authors from [25] proposed a hybrid method between GSA and general algebraic modelling system (GAMS) for determining penetration of solar, wind and hydro power sources. Thanks to the simultaneous integration of these sources combined with reconfiguration, the losses and annual costs were significantly saved for the microgrid system. Besides, the study in [26] used active algorithm of AA to search the place and sizing of DGUs for improving the efficiency of the low voltage microgrid operation with intermittent power outage problem of Iraqi scenario considering maximum condition of load demand. Similarly, another research on the complex Ekbatan residential grid in Tehran, Iran was also implemented [27]. The objective was to determine the appropriate interconnection of components, including different renewable energy sources, in which the uncertainties of irradiance, wind speed, and load were considered to maximize grid reliability with minimum operating costs. Although the above methods have demonstrated their effectiveness in some specific optimization problems, they are still limited in terms of search performance as well as the ability to avoid local traps. To overcome the shortcomings of previous optimization algorithms, in 2018, Pierezan, J., and Coelho, L. D. S. successfully developed an efficient global optimization algorithm, called COA [28]. This algorithm was inspired by the coyotes’ pack behavior in the vast nature. COA has been applied to various optimization problems [29,30], including the problem of determining the optimal penetration of DGUs in various scales of distribution networks [31]. In the study [31], COA is compared with other methods for the reduction of both transmission loss and operational cost of voltage regulators, reaching greater results than others. However, COA also suffers its biggest disadvantage: falling into the local optimal trap in new solution generation techniques in conditions of a large search space and a large number of variables. Thus, several researchers have introduced an improved version called MCOA [7], which has higher stability and efficiency than the original algorithm. For convenience of literature review, a summary of previously published works are presented like Table 1.

**Table 1:** A summary of published studies related to the considered problem.

Applied method	Implemented systems	The considered condition		The objective function			Shortcomings
				Power loss	Voltage deviation	Cost reduction	
		Uncertainty	Harmonics				
APSO [12]	IEEE 33-bus	×	×	✓	✓	×	Unguaranteed convergence. Limited technical constraints.
IA/PSO [13]	IEEE 33-bus, IEEE 69-bus	×	×	✓	✓	×	Poor performance for complex problems. Limited comparison with other methods.
PSO/GSA [14]	IEEE 33-bus, IEEE 69-bus	×	×	✓	✓	✓	High sensitivity to initial parameters. Ignored time-varying load model.
Fuzzy/PSO [15]	IEEE 33-bus	×	×	✓	✓	×	Dependent on the designer’s experience for Fuzzy logic. Small test system.
ABC [16]	IEEE 33-bus, IEEE 69-bus	×	×	✓	✓	×	Long execution time. Lack of comparison with others.
ABC [17]	IEEE 33-bus, IEEE 69-bus, CIGRE grid	×	×	✓	✓	✓	High potential of falling into the local trap. No fair comparison with implemented methods.
ABC [18]	IEEE 34-bus	×	×	✓	✓	✓	Premature Convergence. Small-scale execution system.
CABC [19]	IEEE 38-bus, IEEE 69-bus	×	×	✓	✓	✓	High computational cost. Problem-dependent performance.
BBO [20]	IEEE 33-bus, IEEE 69-bus	×	✓	✓	✓	×	Slow convergence speed. Unconsidered variable load model.
BBO [21]	IEEE 33-bus, IEEE 69-bus	×	✓	✓	✓	×	Unguaranteed performance for high dimensional problems. Limited technical constraints.
SSA [22]	IEEE 33-bus, IEEE 69-bus	×	×	✓	✓	✓	Weak performance for complex problems. Lack of fair comparison with implemented methods.
GWO [23]	IEEE 12-bus, IEEE 33-bus, IEEE 69-bus	×	×	✓	×	×	Premature Convergence. Simple main target.
GA [24]	Microgrid of university in Brazil	×	×	✓	✓	×	Limited balance between exploration and exploitation. Few technical constraints.
GSA/GAMS [25]	IEEE 33-bus	✓	×	✓	×	✓	Complicated algorithm structure. Small-scale test system.
AA [26]	IEEE 33-bus, Baghdad/Al-Ghazalya-655 Microgrid	×	×	✓	×	✓	Limited technical constraints. Limited comparison with other methods.
PSO [27]	The complex Ekbatan residential grid in Iran	✓	×	✓	×	✓	Problem-dependent performance. Small-scale test system.
COA [31]	IEEE 123-bus	×	×	✓	✓	×	Simple targets and constraints. Only consider at a peak load level.

In general, most previous studies have only focused on stabilizing node voltage and reducing line power loss in DSs. However, studies have not adequately considered other important factors. Specifically, aspects such as the initial investment cost of the project and the operating costs throughout the project’s lifespan are often overlooked or only mentioned very briefly. The shortcomings of a comprehensive assessment of these economic factors can reduce the practicality and effectiveness of applying solutions to real-world distribution power grids. In addition, some other authors have also paid attention to the presence of harmonics from nonlinear loads but lacked the calculation of loss and voltage values at all frequency orders [20,21]. Realistically, power loss and voltage exist at both fundamental and higher-order frequencies. Thus, to evaluate the related aspects of installing DGUs correctly, these values should not be limited for computation to the fundamental frequency alone but must also account for higher frequency orders. Considering multiple frequency levels enables a more precise analysis. Furthermore, with the high penetration of renewables such as wind and solar energy sources, it is necessary to comprehensively consider aspects related to technical and economic factors to maximize welfare without affecting the grid [32,33]. Not only that, wind speed and solar irradiance are strongly influenced by natural environmental conditions, leading to continuous and unpredictable variations over time [34]. These fluctuations directly affect the output power of WTDGUs and PVDGUs. As a result, planning these sources into distribution networks becomes more challenging, since their generation capacity cannot always be accurately predicted. Most studies in the past have neglected the uncertainty due to its complexity. Thereby, in this paper, WTDGUs and PVDGUs will be carefully considered to determine the optimal planning strategy under variable conditions of wind speed and solar irradiance, respectively. Moreover, an efficient algorithm for addressing the optimization problem should also be introduced because it has a significant influence on the obtained benefits. The new algorithm with high stability and performance should especially be welcomed and widely used in tackling optimization tasks.

As mentioned, in this study, MCOA, which was published in recent years, is suggested for addressing the optimal installation of WT-PVDGUs with the main goal of minimizing total costs from investment to operation, and also branch loss during project implementation without affecting the existing grid in technical aspects in different-scale power grids, considering strict technical constraints. Overall, the novelties of this study can be briefly indicated as:

(1) This work considers the total costs, including investment cost, OM cost, main grid power import cost for loads, and cost for power losses on distribution branches with strict technical constraints for a long-term project of up to 20 years. This is evaluated as a comprehensive economic consideration while still fully satisfying the stated technical criteria.
(2) This study implements the simultaneous integration of various renewable energy generation sources, such as wind energy and solar energy, into the distribution power grid. Weibull and Beta pdfs with collected actual data over three years are applied to simulate the uncertainties of wind speed and solar radiation for determining the power output of WTDGUs and PVDGUs, respectively. This will contribute to enhancing the accuracy of predicting the output power of grid-connected renewables, and thereby improving the quality of the found solution and reducing the power difference from the actual case.
(3) This work also performs simulations on different scale systems of IEEE 33-bus and 69-bus DSs under harmonic conditions due to the high penetration from nonlinear loads. The IEEE std. 519 for permissible limits of harmonic indices, such as total harmonic distortion (THD) and individual harmonic distortion (IHD), are established. This is also considered a new point due to the complexity of calculations in the frequency domains, and previous works have limited consideration of harmonics.
(4) This research has successfully implemented six powerful algorithms, including EO, TSO, SMA, NGO, COA, and MCOA, to address the same optimization problem. The obtained numerical results are also compared in many aspects of performance and stability to demonstrate the superiority of the suggested method. This also contributes to introducing a powerful optimization method for tackling real-world optimization problems.

The primary contributions of this study are also listed as follows:

(1) From the economic perspective, the study successfully proposed the integrated solution of WTDGUs and PVDGUs for total cost reduction of up to 25.56% and 27.05% for the entire project life cycle on the 33-bus and 69- bus systems, respectively, compared to the original systems. This proves that the appropriate penetration of decentralized generation units has brought great economic benefits through reducing investment cost, OM cost, main grid electricity import cost, and branch power loss cost.
(2) From the technical perspective, the proposed optimal solution not only enhances the bus voltage profile with the weakest voltage from 0.9051 (pu) to 0.9523 (pu) and from 0.9105 (pu) to 0.9558 (pu), but it also reduces the branch current profile with the maximum current from 0.4624 (pu) to 0.3217 (pu) and 0.4902 pu to 0.3441 (pu) for 33-bus and 69-bus DSs, respectively. Additionally, the values represent the influence of harmonics on the grid (THD and IHD), which are also successfully mitigated from (5.7491% and 3.7295%) to (4.2180% and 2.7365%) for the first system and from (5.2646% and 3.4025%) to (4.4783% and 2.8943%) for the second system in the first case. Similarly, in the second case, the reduction of system loss also reached 65.51% and 69.17% for the first and second systems, respectively. This contribution of the proposed solution is great, as it satisfies all the declared technical criteria and significantly improves power quality.

The rest of the paper is divided as follows: Section 2 presents the objective function and technical constraints. Section 3 shows the structure of MCOA. Section 4 describes the application of MOCA to solve the considered problem. Section 5 collects and analyzes simulation results. The last part summarizes the main points of the whole paper, known as the conclusions. In summary, to achieve the main goal of cost-effective saving while still fully meeting the technical criteria of a long-term project for integrating WTDGUs and PVDGUs into the distribution grids, this study has minimized the economic objective function of total combined costs of investment and OM, total cost of importing electricity from the primary grid for loads and total cost of power loss during the grid operation. Technical constraints such as node voltage, line current, harmonic indices, and penetration level are also established, as presented in Section 2.

## 2. Problem formulation

In this work, the optimal solution for the simultaneous connection of wind and solar energy sources to different distribution power grids is determined to maximize economic benefits and satisfy technical constraints.

### 2.1. Objective function

The study integrates WTDGUs and PVDGUs in DSs to reduce the total cost, including 1) the investment and OM costs of WTDGUs and PVDGUs, 2) the power grid purchase cost and 3) the line energy loss cost. Realistically, the total cost of systems before connecting DGUs is the sum of the purchasing energy and energy loss costs. If the integration of DGUs is effective, the total cost will be reduced and smaller than that of the original system. The target in this case can be mathematically expressed as Eq. (1) [7]:


$$\begin{array}{c} Minimize\;{TC}_{DG}=C_{DG}^{Inv\_OM}+C_{DG}^{grid}+C_{DG}^{loss} \end{array}$$ (1)

Where ${TC}_{DG}\;$is total combined cost of the power systems with WTDGUs and PVDGUs; $C_{DG}^{Inv\_OM}$ is the costs from the investment and OM for WT-PVDGUs; $C_{DG}^{grid}$ is the cost of energy purchase through the main grid substation; and $C_{DG}^{loss}$ is the cost of distribution branch energy losses.

#### 2.1.1. Determination of$C_{DG}^{Inv\_OM}$.

$C_{DG}^{Inv\_OM}$is considered as an important component in the objective function and it is the sum of initial capital cost and OM cost. Specifically, the initial capital cost is paid only one time while the OM cost is paid throughout the project life cycle. In other words, the initial capital cost is called fixed cost and the cost of OM is called variable cost which dependents on the power output. The $C_{DG}^{Inv\_OM}$ can be determined by Eq. (2):


$$\begin{array}{c} \;C_{DG}^{Inv\_OM}={FC}_{DG}+{VC}_{DG} \end{array}$$ (2)

Where ${FC}_{DG}$ and ${VC}_{DG}$ are respectively fixed cost and variable cost that can be calculated using Eqs. (3)-(4) [35]:


$$\begin{array}{c} {\;FC}_{DG}=\sum_{w=\text{1}}^{N_{WT}}\left(C_{WT}^{Cap}{.P}_{WT,w}^{ins}\right)+\sum_{p=\text{1}}^{N_{PV}}\left(C_{PV}^{Cap}{.P}_{PV,p}^{ins}\right)\; \end{array}$$ (3)

$$\begin{array}{c} {\;VC}_{DG}=\text{9}\text{1}.\text{2}\text{5}\times \left[\sum_{y=\text{1}}^{N_{y}}\sum_{h=\text{1}}^{\text{9}\text{6}}\sum_{w=\text{1}}^{N_{WT}}\left({\rho_{y}.C}_{WT}^{O\&M}{.P}_{WT,w,h,y}^{ope}\right)+\sum_{y=\text{1}}^{N_{y}}\sum_{h=\text{1}}^{\text{9}\text{6}}\sum_{p=\text{1}}^{N_{PV}}\left(\rho_{y}.C_{PV}^{O\&M}{.P}_{PV,p,h,y}^{ope}\right)\;\right] \end{array}$$ (4)

As mentioned, this work supposes the project period of 20 years considering the uncertain characteristic of wind speed and solar irradiance over four seasons. In Eq. (4), 96 is the number of hours for four days in which each day is a representative for each season. Besides, coefficient of 91.25 is used to convert 96 hours into 8760 hours (i.e., the result of (91.25×96) is 8760 hours) [36]. Moreover, annual cost which is found from Eq. (4), is not the same for the considered *y*^*th*^ year due to the impact of $\rho_{y}$. Specifically, $\rho_{y}$is a function of the year order, and its annual value can be identified as Eq. (5) [7]:


$$\begin{array}{c} {\;\rho }_{y}={\left(\frac{\text{1}}{\text{1}+ir}\right)}^{y} \end{array}$$ (5)

where, *ir* is defined as the interest rate (*ir* = 0.09); and *y* is the year index of the 20-year project.

#### 2.1.2. Determination of$C_{DG}^{grid}$.

By installing both WTDGUs and PVDGUs in the distributions system, each system have three main power sources including 1) distribution transformers connected at the slack bus, 2) WTDGUs and 3) PVDGUs. The imported power energy cost $C_{DG}^{grid}\;$in Eq. (1) is the amount of money paid for the electric company from using energy supplied through the distribution transformer. This cost is computed by using Eq. (6) [37]:


$$\begin{array}{c} {\;C}_{DG}^{grid}=\text{9}\text{1}.\text{2}\text{5}.{Price}^{grid}.\sum_{y=\text{1}}^{N_{y}}\sum_{h=\text{1}}^{N_{h}}\sum_{l=\text{1}}^{N_{ld}}\left(\rho_{y}.P_{DG,l,h,y}^{ld}\right)\; \end{array}$$ (6)

Where $P_{DG,l,h,y}^{ld}$ (in MW) is the real power of the *l*^*th*^ load at the *h*^*th*^ hour of the *y*^*th*^ year after connecting WT-PVDGUs; and ${Price}^{grid}$ (in $/MWh) is price of energy buying from primary grid.

#### 2.1.3. Determination of$C_{DG}^{loss}$.

Current flowing in distribution lines is a main factor affecting economic as well as the technical issues of distribution systems [38]. The current on branches with the high magnitude value can lead to high active power loss and the transmission power congestion [39]. The energy loss cost for one hour can be insignificant but that over one year and many years is serious. Therefore, the energy loss cost should be considered as a primary economic issue that needs to be minimized as much as possible. This cost can be determined using Eq. (7):


$$\begin{array}{c} {\;C}_{DG}^{loss}=\text{9}\text{1}.\text{2}\text{5}.{Price}^{loss}.\sum_{y=\text{1}}^{N_{y}}\sum_{h=\text{1}}^{\text{9}\text{6}}\sum_{bh=\text{1}}^{N_{bh}}\left(\rho_{y}{.P}_{DG,bh,h,y}^{ls}\right)\;(\$) \end{array}$$ (7)

Where $P_{DG,bh,h,y}^{ls}$ (in MW) is the *bh*^*th*^ branch power loss at the *h*^*th*^ hour in the *y*^*th*^ year with installing WT-PVDGUs; ${Price}^{loss}\;$(in $/MWh) is energy loss price.

In this paper, harmonic flows are produced by the nonlinear loads in the DSs and so, harmonic distortions are considered for calculating the active power loss, $P_{DG,bh,h,y}^{ls}$ by applying Eq. (8) [20]:


$$\begin{array}{c} P_{DG,bh,h,y}^{ls}=\text{3}.\sum_{bh=\text{1}}^{N_{bh}}\left(I_{DG,\;bh,h,y}^{\text{2}}.R_{bh}\right) \end{array}$$ (8)

Where $I_{DG,bh,h,y}$ of the *bh*^*th*^ branch current at the *h*^*th*^ hour from the *y*^*th*^ year with installing WT-PVDGUs and it can be obtained by using Eq. (9) [40]:


$$\begin{array}{c} {\;I}_{DG,bh,h,y}=\sqrt{{\left(I_{DG,bh,h,y,\text{1}}\right)}^{\text{2}}+\sum_{hr=\text{2}}^{Hr}{\left(I_{DG,bh,h,y,hr}\right)}^{\text{2}}} \end{array}$$ (9)

### 2.2. Constraints

The mentioned economic objective should be minimized as much as possible, considering the following technical constraints:

#### 2.2.1. The power balance constraints.

To maintain frequency and voltage stability, the total generation should be equal to the total power consumption, including power losses [40]. After placing WTDGUs and PVDGUs in distribution systems, the total generation is the sum of power supplied by the primary grid and the power generated by WTDGUs and PVDGUs. Thus, the power balance equations are expressed by Eqs. (10)-(11) [27]:


$$\begin{array}{c} {\;P}_{DG,h,y}^{grid}+\sum_{w=\text{1}}^{N_{WT}}P_{WT,w,h,y}^{ope}+\sum_{p=\text{1}}^{N_{PV}}P_{PV,p,h,y}^{ope}=\sum_{bh=\text{1}}^{N_{bh}}P_{DG,bh,h,y}^{ls}+\sum_{l=\text{1}}^{N_{lo}}P_{DG,l,h,y}^{ld} \end{array}$$ (10)

$$\begin{array}{c} Q_{DG,h,y}^{grid}+\sum_{w=\text{1}}^{N_{WT}}Q_{WT,w,h,y}^{ope}+\sum_{p=\text{1}}^{N_{PV}}Q_{PV,p,h,y}^{ope}=\sum_{bh=\text{1}}^{N_{bh}}Q_{DG,bh,h,y}^{ls}+\sum_{l=\text{1}}^{N_{lo}}Q_{DG,l,h,y}^{ld} \end{array}$$ (11)

In the Eq. (11), ${\text{Q}}_{\text{WT},\text{w},\text{h},\text{y}}^{\text{ope}}$ and $Q_{\text{PV},w,h,y}^{ope}$ can be found by:


$$\begin{array}{c} Q_{WT,w,h,y}^{ope}=P_{WT,w,h,y}^{ope}.\alpha_{WT,w};\text{ Where},\;{\;\alpha }_{WT,w}=\pm \text{tan}({cos}^{-\text{1}}({PF}_{WT,w})) \end{array}$$ (12)

$$\begin{array}{c} {\;Q}_{PV,p,h,y}^{ope}=P_{PV,p,h,y}^{ope}.\alpha_{PV,p};\text{ Where},\;\alpha_{PV,p}=\pm \text{tan}({cos}^{-\text{1}}({PF}_{PV,p})) \end{array}$$ (13)

where, ${{PF}_{WT,w}\text{ and }PF}_{PV,p}\;$are the power factor values of the *w*^*th*^ inverter-based WTDGU and the *p*^*th*^ inverter-based PVDGU, and these values should be within 0 and 1 [41].

#### 2.2.2. The branch current limit.

The penetration of renewable energy sources into DSs can lead to increase or decrease in the current on the distribution line. Therefore, to keep the original configuration of the grid, the current with WT-PVDGUs must not exceed the following allowable limit as Eq. (14) [42]:


$$\begin{array}{c} I_{DG,bh}\leq I_{bh}^{Max};\;bh=\text{1},\;\text{2},\;\ldots ,\;N_{br} \end{array}$$ (14)

#### 2.2.3. The bus voltage limits.

The load voltage after adding renewable power sources in DSs will change compared to the initial values. According to the BS EN 50160 standard, all loads should be operated within the range [0.90, 1.10] pu. However, in this study, the voltage is limited to a more stringent range of 0.95 to 1.05 pu [31]. The mathematical equation is presented as Eq. (15):


$$\begin{array}{c} {\;V}^{Min}\leq V_{DG,i,h,y}\leq V^{Max},\;i=\text{1},\;\text{2},\;\ldots ,\;N_{bs} \end{array}$$ (15)

where, $V^{Max}$ and $V^{Min}$ are allowable maximum and minimum voltage limits for all loads, respectively. $V_{DG,i,h,y}$ is the operation voltage of load for the *i*^*th*^ bus in the *h*^*th*^ hour from the *y*^*th*^ year. This voltage value can be obtained by using Eq. (16) [40]:


$$\begin{array}{c} {\;V}_{DG,i,h,y}=\sqrt{{\left(V_{DG,i,h,y}^{\text{1}}\right)}^{\text{2}}+\sum_{hr=\text{2}}^{Hr}{\left(V_{DG,i,h,y,hr}\right)}^{\text{2}}} \end{array}$$ (16)

#### 2.2.4. The harmonic voltage distortion constraints.

The presence of harmonic distortions can lead to various undesired effects, such as increased current in distribution lines, additional energy losses, and temperature rise in electric components [43]. These can reduce the life and damage grid-connected equipment. Therefore, IEEE Std. 519 is applied with maximum allowable limits of 3% and 5% for individual and total voltage harmonic distortions, respectively, and the harmonic indices are set in the constraints like Eqs. (17, 18) [20]:


$$\begin{array}{c} {IHD}_{V,i,h,y}^{hr}\leq {\;IHD}_{V}^{Max};\;i=\text{1},\;\text{2},\;\text{3}\ldots ,\;N_{bs} \end{array}$$ (17)

$$\begin{array}{c} {\;THD}_{V,i,h,y}\leq {\;THD}_{V}^{Max};\;i=\text{1},\;\text{2},\;\text{3}\ldots ,\;N_{bs} \end{array}$$ (18)

Where, ${IHD}_{V,i,h,y}^{hr}$ and ${THD}_{V,i,h,y}$ are determined by using Eq. (19) and Eq. (20), respectively:


$$\begin{array}{c} \;{IHD}_{V,i,h,y}^{hr}\;=\left[\frac{V_{DG,i,h,y,hr}}{V_{DG,i,h,y}^{\text{1}}}\right].\text{100} \end{array}$$ (19)

$$\begin{array}{c} {\;THD}_{V,i,h,y}\;=\left[\frac{\sqrt{\sum_{hr=\text{2}}^{Hr}{\left(V_{DG,i,h,y,hr}\right)}^{\text{2}}}}{V_{DG,i,h,y}^{\text{1}}}\right].\text{100} \end{array}$$ (20)

#### 2.2.5. The WT-PVDGU’s penetration level constraints.

In this study, the total actual generation of all connected units should not exceed the sum of the total consumption at each time. To express the generation limit in mathematical model, the equation is suggested as Eq. (21) [8]:


$$\begin{array}{c} \;\sum_{y=\text{1}}^{N_{y}}\sum_{h=\text{1}}^{\text{9}\text{6}}\sum_{w=\text{1}}^{N_{WT}}P_{WT,w,h,y}^{ope}\;+\sum_{y=\text{1}}^{N_{y}}\sum_{h=\text{1}}^{\text{9}\text{6}}\sum_{p=\text{1}}^{N_{PV}}P_{PV,p,h,y}^{ope}\leq \sum_{y=\text{1}}^{N_{y}}\sum_{h=\text{1}}^{\text{9}\text{6}}\sum_{l=\text{1}}^{N_{lo}}P_{ld,l,h,d}+\sum_{y=\text{1}}^{N_{y}}\sum_{h=\text{1}}^{\text{9}\text{6}}\sum_{bh=\text{1}}^{N_{bh}}P_{ls,bh,h,y} \end{array}$$ (21)

The installed capacity of each connected unit is also limited within the predetermined ranges as Eqs. (22) and (23):


$$\begin{array}{c} P_{WT}^{Min}\leq P_{WT,w}^{inst}\leq P_{WT}^{Max};\;w=\;\text{1},\;\text{2},\;\text{3}\;\ldots ,\;N_{WT} \end{array}$$ (22)

$$\begin{array}{c} {\;P}_{PV}^{Min}\leq P_{PV,p}^{inst}\leq P_{PV}^{Max};\;p=\;\text{1},\;\text{2},\;\text{3}\;\ldots ,\;N_{PV} \end{array}$$ (23)

### 2.3. Uncertainty modeling

In this study, a considered year includes four different seasons, and the representative day of each season is calculated to simulate the variable behavior of the wind turbine and photovoltaic distributed generation units. Each representative day is divided into 24-hour segments, resulting in a total of 96 time segments for a whole year. To predict the hourly wind speed and solar irradiance, the Weibull and Beta pdfs are utilized, based on historical data collected over three years from the actual research site. Additionally, the load profile is assumed to adhere to the IEEE RTS-96 [44].

#### 2.3.1. Calculation of wind power uncertainty.

For simulating wind speed, in the Weibull pdf, two important parameters deciding the shape of the graph are the scale index (sc) and the shape index (sh) [45, 46]. In previous studies [44,47,48], sh = 2 and $sc=\text{1}.\text{1}\text{2}\text{8}.s_{m}$ (where $s_{m}$ is average of wind speed) were proposed for estimating probability distribution of wind speed. And Weibull pdf becomes the Rayleigh pdf as expressed in Eq. (24) [47]:


$$\begin{array}{c} {\;W}_{r}(s)=\frac{\text{2}.s}{{\left(\text{1}.\text{1}\text{2}\text{8}.s_{m}\right)}^{\text{2}}}.\text{exp}\left[-{\left(\frac{s}{\text{1}.\text{1}\text{2}\text{8}.s_{m}}\right)}^{\text{2}}\right];\;\text{0}<s<\infty \end{array}$$ (24)

In this study, the wind speed values expected in the specified location are assumed to fall within 20 intervals of wind speed, with each step of 1.0 m/s as supposed. To determine the probability of wind speed at a specific hour, the model of Eq. (25) is applied [44]:


$$\begin{array}{c} y_{w}(s)=\int_{s_{v\text{1}}}^{s_{v\text{2}}}W_{r}(s).ds \end{array}$$ (25)

where, $s_{v\text{1}}$and $s_{v\text{2}}$ are wind speed limits of stage s.

Additionally, to calculate the actual power output of wind turbines for each particular wind speed, the function as Eq. (26) is used to depict the generation characteristic of the wind turbine [45]:


$$\begin{array}{c} {\;P}_{WT}(s)=\left\{ \begin{array}{c} @l\;\text{0}\;if\;s<s_{in}\;or\;s>s_{out}\;\\ P_{r}.\left(\frac{s-s_{in}}{s_{r}-s_{in}}\right),\;if\;s_{in}\leq s\leq \;s_{r}\\ P_{r},\;if\;s_{r}<s\leq \;s_{out}\; \end{array}\right. \end{array}$$ (26)

By using the probability function, the expected output power from the wind turbine can be reached as shown in Eq. (27). And finally, we can find the expected total output power at a specific time period by applying Eq. (28) [48].


$$\begin{array}{c} {\;Po}_{WT}(s)=P_{WT}(s).y_{w}(s) \end{array}$$ (27)

$$\begin{array}{c} {Po}_{WT}^{total}=\int_{\text{0}}^{\infty }{Po}_{WT}(s).ds \end{array}$$ (28)

#### 2.3.2. Calculation of solar power uncertainty.

In this study, the Beta pdf is used to simulate the solar irradiance at every hour [44], and its equation can be described as Eq. (29) [48, 49]:


$$\begin{array}{c} S_{b}(r)=\left\{ \begin{array}{c} @c\frac{\Gamma (a+b)}{\Gamma (a)\Gamma (b)}r^{a-\text{1}}(\text{1}-r{)}^{b-\text{1}},\;\text{if }\text{0}\leq r\leq \text{1},\;\;a\geq \text{0},\;\;b\geq \text{0}\\ \text{0}\text{ otherwise} \end{array}\right. \end{array}$$ (29)

where, Γ(·) is defined as the gamma function. Two parameters, *a* and *b*, can be found by using the standard deviation and the mean of solar irradiance [32].

In this case, solar irradiance is assumed to consist of 20 irradiance intervals in the considered range, with each step of 0.05 kW/m^2^. The probability of solar irradiance at a specific hour can be determined as Eq. (30) [44]:


$$\begin{array}{c} {\;y}_{p}(r)=\int_{r_{s\text{2}}}^{r_{s\text{1}}}S_{b}(r).dr \end{array}$$ (30)

where, $r_{s\text{1}}$and $r_{s\text{2}}$ are solar irradiance limits of state r.

Besides, the output power of photovoltaic distributed generation unit corresponding to each specific solar irradiance (r) can be found by using Eq. (31) [49].


$$\begin{array}{c} P_{PV}(r)=FF.N.V(r).I(r) \end{array}$$ (31)

Where, FF and N are the fill factor and the number of photovoltaic modules; and V(r) and I(r) are voltage and current of photovoltaic module with the solar radiation *r*, respectively. The calculation of these four factors can be referred to the study [49].

Lastly, the expected output power of the photovoltaic unit considering the effect from solar irradiance is obtained by applying Eq. (32), and the expected total output power of the photovoltaic module at a specific time period can be calculated by using Eq. (33) [48,50]:


$$\begin{array}{c} {\;Po}_{PV}(r)=P_{PV}(r).y_{p}(r) \end{array}$$ (32)

$$\begin{array}{c} {\;Po}_{PV}^{total}=\int_{\text{0}}^{\text{1}}{Po}_{PV}(r)dr \end{array}$$ (33)

## 3. Applied optimization algorithm

In this study, a robust method called MCOA is applied to address the problem of optimal installation of WTDGUs and PVDGUs in distribution networks. MCOA is a modified version of the original algorithm, COA, which has been demonstrated to have many outstanding advantages over other methods in solving a variety of optimization problems. The main advantages of COA that can be listed are (1) the good balance between exploration and exploitation of potential solutions, (2) the strong ability to maintain high diversity in the population to find quality solutions by avoiding premature convergence, (3) the great performance and stability in determining the global optimal solution and (4) the found solution quality is superior to others in the optimization fields [51–53]. Although COA is a powerful algorithm, it still has disadvantages like time-consuming data processing, low performance, and stability when solving complex problems with a large number of variables. Therefore, MCOA was developed to inherit the advantages and overcome the disadvantages of the original algorithm. Like the original COA, each individual (coyote) in the population from MCOA is also characterized by living conditions and the quality of living conditions. The living condition corresponds to the solution of the optimization task, and the quality of the living condition reports the fitness level of each solution. In this algorithm, the coyote community is separated into $N_{gr}$ groups, and each group has $N_{co}\;$coyotes. Thus, the coyote population $\left(N_{po}\right)$ is equal to ($N_{gr}\times N_{co}$). The structure of MCOA can be presented as follows [7]:

Like other population-based algorithms, the initial population is randomly produced within the constraints of the search space for possible solutions to the problem, like Eq. (34):


$$\begin{array}{c} F_{g,c}=F^{Min}+r_{d}.\left(F^{Min}+F^{Max}\right);\;g=\text{1},\;\text{2},\;\text{3}\ldots \;N_{gr}\;\&\;c=\;\text{1},\;\text{2},\;\text{3}\ldots \;N_{co} \end{array}$$ (34)

where, $F^{Max}$ and $F^{Min}$ are maximum and minimum allowable values of each control variable in the proposed solution. In this algorithm, there are two main stages for creating the next generation. In the first-generation stage, MCOA uses the following mathematical equation for updating new solutions [7]:


$$\begin{array}{c} \;F_{g,c}^{New}=F_{g,c}+r_{d}.\left(F_{best,g}-F_{r\text{1},g}\right)+r_{d}.\left(F_{Gbest}-F_{r\text{2},g}\right);\;g=\text{1},\;\text{2},\;\text{3}\ldots \;N_{gr}\;\&\;c=\;\text{1},\;\text{2},\;\text{3}\ldots \;N_{co} \end{array}$$ (35)

The variables in the newly obtained solutions are compared to the allowable values of $F^{Max}$ and $F^{Min}$. Variables with a higher value than the maximum allowable value of $F^{Max}$ are set to the maximum value, and vice versa; variables with a smaller value than the minimum allowable value of $F^{Min}\;$are set to the minimum value. Each created new solution is re-evaluated according to the fitness function $\left({FF}_{g,c}^{New}\right)$, and the comparison is executed between the old and new solutions to keep the more dominant solution and its fitness.

In the second-generation stage of producing new solution, only one new solution ($F_{g}^{New}$) is produced for each group by using Eq. (36) or Eq. (37).


$$\begin{array}{c} {\;F}_{g}^{New}=F_{Gbest}+r_{d}.\left(F_{Gbest}-F_{best,r\text{1}}\right)+r_{d}.\left(F_{Gbest}-F_{best,r\text{2}}\right) \end{array}$$ (36)

$$\begin{array}{c} \;F_{g}^{New}=F_{Gbest}+r_{d}.\left(F_{Gbest}-F_{best,r\text{1}}\right)+r_{d}.\left(F_{Gbest}-F_{best,r\text{2}}\right)+r_{d}.\left(F_{Gbest}-F_{best,r\text{3}}\right) \end{array}$$ (37)

$F_{best,r\text{1}},F_{best,r\text{2}}\text{ and }F_{best,r\text{3}}$are the best solutions of groups, and they are chosen randomly. The two equations have the same manner that applies the search space around the global solution to find new solutions, but they are different in using the number of increased steps. Eq. (36) uses two increased steps while Eq. (37) needs one more increased step. Thus, the application condition of the two equations is very important for finding a more effective solution than the current global solution. To have a suitable selection, a ratio between the number of close solution couple numbers (*N*_*cc*_) and the maximum solution couple number (*N*_*mc*_) is determined, and then it is compared to a predetermined threshold (*ε*). If (*N*_*cc*_*/ N*_*mc*_) is smaller than the *ε* value, Eq. (36) is applied. For another case (i.e. *N*_*cc*_*/ N*_*mc*_ is equal to or higher than the *ε* value), Eq. (37) is selected [7].

## 4. Using proposed algorithm to solve the considered problem

### 4.1. Initialization for solutions

For producing the initial population as presented in Eq. (34), the boundaries of $F^{Max}$ and $F^{Min}$ are defined as follows:


$$\begin{array}{c} F^{Max}=[{Po}_{n}^{Max},{Si}_{n}^{Max}\;];\;n=\;\text{1},\;\text{2},\;\text{3}\ldots \;N_{DG} \end{array}$$ (38)

$$\begin{array}{c} F^{Min}=[{Po}_{n}^{Min},{Si}_{n}^{Min}\;];\;n=\;\text{1},\;\text{2},\;\text{3}\ldots \;N_{DG} \end{array}$$ (39)

Once the solutions are generated, the forward-backward sweep technique (FW-BWST) [54] is applied for analysis of the power flow and the harmonic flow on all distribution lines. Based on obtained components (including branch current, bus voltage, total and individual harmonic voltage distortion violations) and their allowable limits, penalty terms for each proposed solution are calculated. Finally, the fitness function can be identified from the obtained results of the objective function and the penalty function as Eq. (40).


$$\begin{array}{c} {\;FF}_{g,c}={OF}_{g,c}+\omega_{I}.\sum_{bh=\text{1}}^{N_{bh}}{\Delta I}_{DG,bh,g,c}^{\text{2}}+\omega_{V}.\sum_{i=\text{1}}^{N_{bs}}{\Delta V}_{DG,i,g,c}^{\text{2}}+\omega_{THD}.\sum_{i=\text{1}}^{N_{bs}}{\Delta THD}_{V,i,g,c}^{\text{2}}+\omega_{IHD}.\sum_{i=\text{1}}^{N_{bs}}{\Delta IHD}_{V,i,g,c}^{\text{2}} \end{array}$$ (40)

### 4.2. Generating and handling new solutions

As presented in Section 3, the suggested method, MCOA performs two generation stages for finding potential solutions, in which, new solutions in the first stage ($F_{g,c}^{New}$) are produced by using Eq. (35) and the new solution in the second stage ($F_{g}^{New}$) are generated by using Eq. (36) or Eq. (37). New solutions will be checked for determining the limit violation, if any variable in the produced solution falls outside the acceptable limits, it will be brought back to the nearest boundary limit.

### 4.3. The MCOA flowchart for solving the optimization problem of integrating WT-PVDGUs

**Fig 1:**
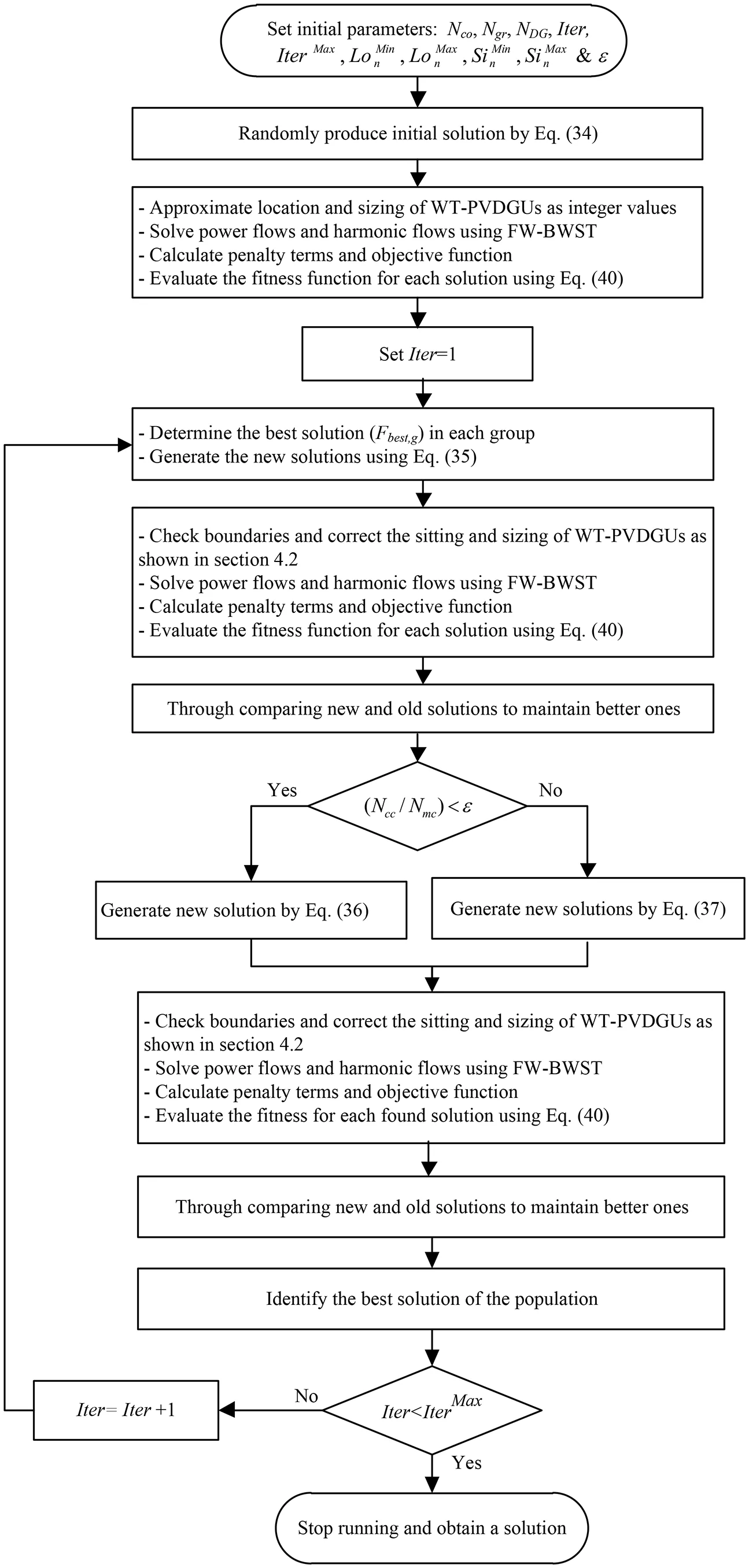
The MCOA flowchart for optimizing the sitting and sizing of WT-PVDGUs.

An iterative algorithm by using MCOA is repeatedly implemented for finding the most suitable solution of installation of WT-PVDGUs until reaching the predetermined maximum iteration (${Iter}^{Max}$). Applying MCOA for the considered optimization problem is briefly summarized in Fig 1.

## 5. Simulation results

### 5.1. Case 1: Considering the uncertainty of DGUs

In this paper, the six optimization algorithms, such as EO, TSO, SMA, NGO, COA and MCOA, are applied to find optimal connection of WT-PVDGUs in IEEE 33-bus and IEEE 69-bus DSs, considering the strict technical constraints. All algorithms are implemented in MATLAB (ver. 2017) on a PC with 8.0 RAM and a 1.8 GHz processor. For suggested method, the population and iteration numbers are surveyed to select the appropriate values. Specifically, *N*_*co*_ is equal to *N*_*gr*_ as 4. The *ε* value is chosen to be 0.2 as the result from the survey in the range of [0.0, 1.0] with the step size of 0.2 [7]. In addition, the remaining specific parameters of the used methods are selected according to the recommendations by previous studies such as EO [55], TSO [56], SMA [57], NGO [58] and COA [28]. Moreover, parameters related to calculating grid operational costs are also reported in Table 2.

**Table 2:** The information of calculating the related costs.

Item	Value
$C_{WT}^{Cap}\text{ and }C_{WT}^{O\&M}$ [59]	1,882,000 $/MW and 10 $/MWh
$C_{PV}^{Cap}\text{ and }C_{PV}^{O\&M}$ [37]	770,000 $/MW and 10 $/MWh
${Price}^{loss}\text{ and }{Price}^{grid}$ [37]	60 $/MWh and 96 $/MWh

As mentioned, this study applies the Weibull and Beta pdfs for estimating the probability distribution of wind speed and solar radiation. The Weibull and Beta pdfs have been widely used in previous studies and have proven effective in predicting wind speed and solar radiation, respectively. For calculating the probability distribution of wind speed and solar radiation, actual measurement data at the study area with the coordinates of 56.4907^o^ N and 4.2026^o^ W (Scotland) are obtained through using POWER Data Access Viewer v2.0.0 [60]. In addition, to improve the accuracy in predicting wind speed and solar radiation, data for three consecutive years from 2018 to 2020 are collected. The concerned period is one year, separated into four seasons, in which each season is represented by one day with 24 one-hour intervals. Thus, the mean wind speed as well as the mean solar radiation and its standard deviation of representative days are reported in S3, S4 and S5 Tables in S1 File. Technical parameters of the wind turbine and photovoltaic module are also shown in S6 Table in S1 File [61] and S7 Table in S1 File [44] in the Supplementary Material, respectively. For modeling load demand in test systems, load factors of IEEE RTS-96 are used and presented in S8 Table in S1 File [44]. In this case, three PVDGUs and three WTDGUs are considered for connection to systems, with the minimum and maximum numbers for each connection location supposed to be in the limits of (01 wind turbine and 15 wind turbines) and (1,000 photovoltaic modules and 20,000 photovoltaic modules). The rated power of each wind turbine and each photovoltaic module is 100 kW and 75 W, respectively, and the inverters of these units are also assumed to be able to work at the lagging power factor of 0.9, as in previous studies [36,62]. As stated, the study examines the occurrence of harmonic distortions on the power grid. Harmonics can significantly affect the performance and reliability of grid-connected components such as transformers, capacitors, etc. When nonlinear loads introduce harmonic currents into the network, the distorted waveform increases the current flowing through the electrical equipment. For transformers, harmonics can lead to increased losses, overheating, insulation ageing, and reduced service life [63]. Besides, capacitors are also affected by harmonics. Capacitors tend to draw harmonic currents, which can lead to overcurrent and increased dielectric stress. In some cases, resonance that amplifies harmonics also increases voltage distortion [64]. In addition to directly impacting individual equipment, harmonics also contribute to increased network losses on distribution lines [65]. For these reasons, assessing harmonic distortions by considering harmonic criteria is essential to maintaining power quality and ensuring reliable system operation in modern power systems with the increasing use of power electronics. Therefore, this study evaluates the impact of harmonics, which are produced by nonlinear loads, with the harmonic spectrum, including harmonic magnitude and phase angle, as referenced from [66], as given in Table 3. Therefore, FW/BWST is applied to address power flow and harmonic flow problems in all test systems [54]. Firstly, at the fundamental frequency, the power flow is computed to find node voltage and line power loss, and then, at each higher frequency order, the admittance matrix is determined according to the frequency. Finally, the node voltages and line losses at the considered frequency orders are calculated based on the corresponding admittance matrices and harmonic current injections. From the obtained results, harmonic indices, including THD and IHD, can be determined by using the above Eqs. (19) and (20).

**Table 3:** The harmonic spectrum.

Harmonic order	Magnitude	Angle
5, 7, 11, 13, 17	76.5%, 62.7%, 24.8%, 12.7%, 7.1%	28^o^, -180^o^, -59^o^, 79^o^, -253^o^

#### 5.1.1. The simulation results for IEEE 33-bus DS.

**Fig 2:**
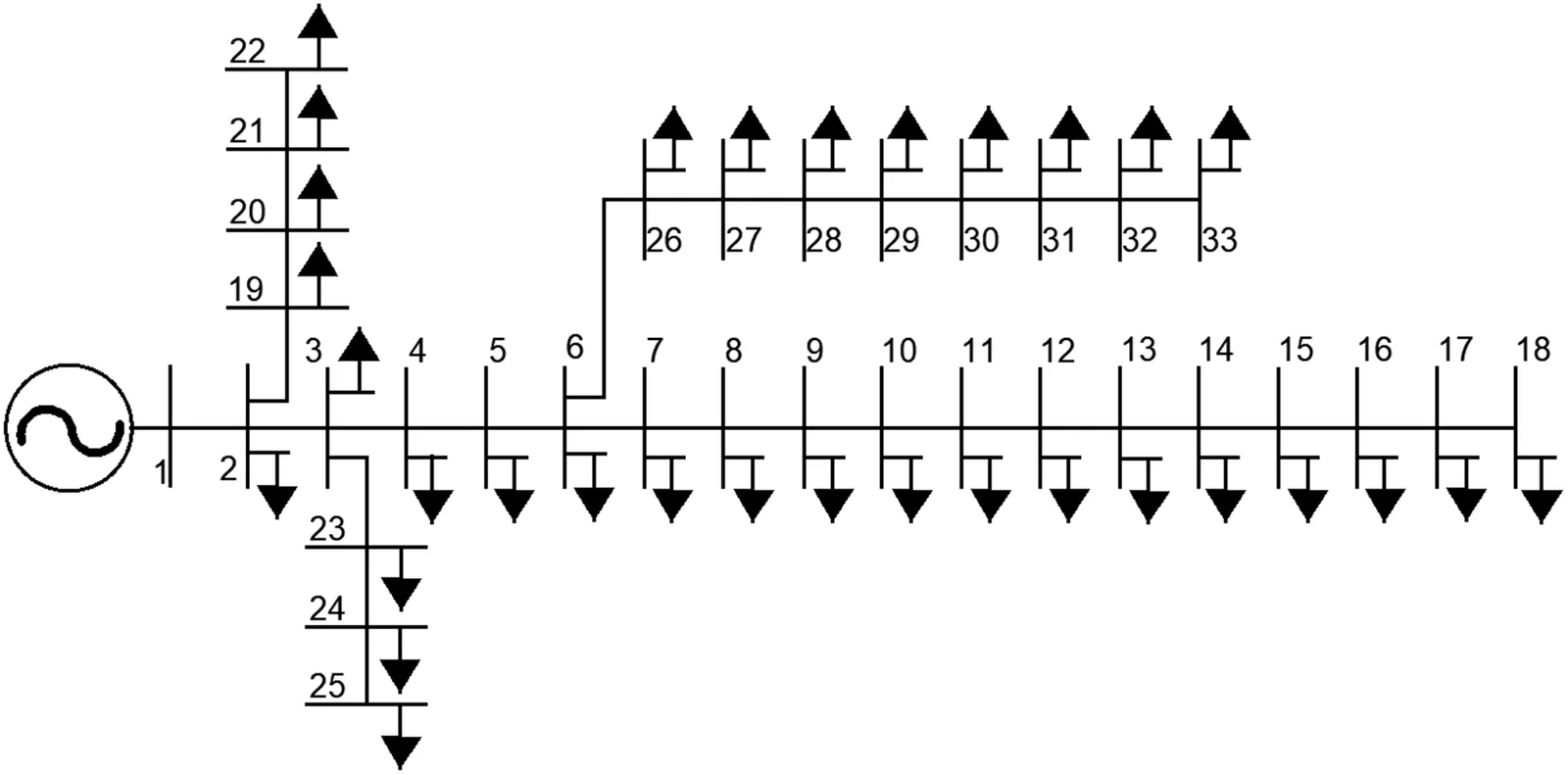
IEEE 33-bus DS.

In this section, IEEE 33-bus DS is used as a test system to find the optimal solution of the placement of WTDGUs and PVDGUs. The system’s configuration is shown in Fig 2, and its data are shown in S1 Table in S1 File [9], and five nonlinear loads are located at buses No. 15, 20, 24, 29, and 32.

**Fig 3:**
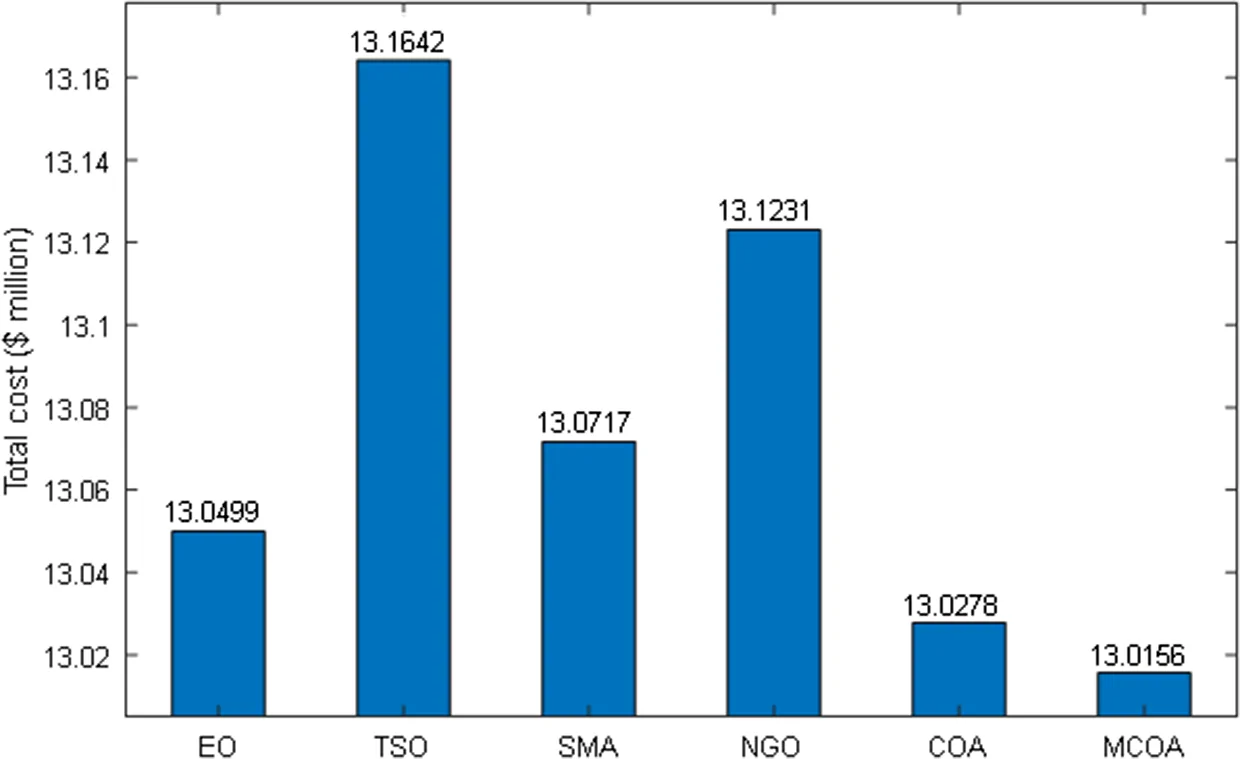
The comparison of the best total cost for operating system in 33-bus system.

The best total cost from the implemented methods of EO ($13.0499 million), TSO ($13.1642 million), SMA ($13.0717 million), NGO ($13.1231 million), COA ($13.0278 million) and MCOA ($13.0156 million) in 40 trials is presented as six bars in Fig 3. Obviously, the bar of the suggested method, MCOA is the shortest with $13.0156 million, and its value is less than those of EO, TSO, SMA, NGO and COA by $34.3 thousand, $148.6 thousand, $56.1 thousand, $107.5 thousand and $12.2 thousand, respectively. In other words, the total cost difference between the compared methods (EO, TSO, SMA, NGO, and COA) and the suggested method is equivalent to 0.26%, 1.14%, 0.43%, 0.82% and 0.094%. Although these differences are not significant, they contribute to reflecting the effectiveness of MCOA compared to the original method and others. Moreover, to clearly show the economic benefit from integrating WT-PVDGUs into the grid compared to the base system, the obtained results have also been presented in Table 4. Before connecting WT-PVDGUs, the total cost (${TC}_{DG}$) at the original system, including energy loss cost on branches ($388.695 thousand) and energy purchase cost for loads ($17.3329 million) over 20 years, is $17.7217 million. However, thanks to WT-PVDGUs being integrated into the system by using the applied algorithms, the total cost from the found optimal solutions is much lower than that of the base system. Specifically, MCOA sharply cut the total cost from $17.7217 million to $13.0156 million with the reduction of $4.7060 million, corresponding to 26.56% in cost savings. The cost saving values compared to the base case from the remaining methods are not as good as that of MCOA, but it is still significant. In detail, it ranges from 25.72% (saving to $4.5574 million) at the worst method (TSO) to 26.48% (saving to $4.6938 million) at the second-best method (COA). This demonstrates the huge economic benefit from the proper connection of WTDGUs and PVDGUs in the distribution network.

**Table 4:** The comparison of cost components in the total cost of implemented methods in 33-bus system.

Method	Base system	EO	TSO	SMA	NGO	COA	MCOA
Cost of the investment, OM (${TC}_{DG}^{Inv\_OM},\;$$ million)	0	6.3621	6.1816	6.3591	6.2410	6.3714	6.3884
Cost of purchasing energy from the main grid (${TC}_{DG}^{grid},\;$$ million)	17.3329	6.5764	6.8583	6.5812	6.7655	6.5619	6.5353
Cost of energy loss (${TC}_{DG}^{loss},\;$$ thousand)	388.695	111.398	124.417	131.515	116.645	94.540	91.830
Total cost (${TC}_{DG},\;$$ million)	17.7217	13.0499	13.1642	13.0717	13.1231	13.0278	13.0156
Saving cost ($ million) =$TC-{TC}_{DG}$	–	4.6717	4.5574	4.6499	4.5985	4.6938	4.7060
Saving cost (%)	–	26.36	25.72	26.24	25.95	26.48	26.56

All costs related to the objective function are also analyzed specifically in Table 4. The costs (${TC}_{DG}^{Inv\_OM}$ and ${TC}_{DG}^{grid}$) from the solution of MCOA, EO, TSO, SMA, NGO and COA are ($6.3884 million and $6.5353 million), ($6.3621 million and $6.5764 million), ($6.1816 million and $6.8583 million), ($6.3591 million and $6.5812 million), ($6.2410 million and $6.7655 million), and ($6.3714 million and $6.5619 million), respectively. Obviously, the costs of investment and OM from MCOA are higher than the five compared methods, but the cost of purchasing electricity from the main grid of MCOA is more economical than all other methods. Besides, the cost (${TC}_{DG}^{loss}$) from MCOA ($91.830 thousand) is also lower than EO ($111.398 thousand), TSO ($124.417 thousand), SMA ($131.515 thousand), NGO ($116.645 thousand), and COA ($94.540 thousand). In other words, the optimal solution of the suggested method has higher investment and OM costs, but it brings better economics compared to other methods in terms of electricity import cost and power loss cost. As mentioned, the most important value is the total cost for implementing this project, and MCOA has found the best solution in the economic aspect, considering technical requirements, compared to others. Therefore, it can be affirmed that MCOA is the most effective method for the optimization problem in this case.

#### 5.1.2. The simulation results for IEEE 69-bus DS.

**Fig 4:**
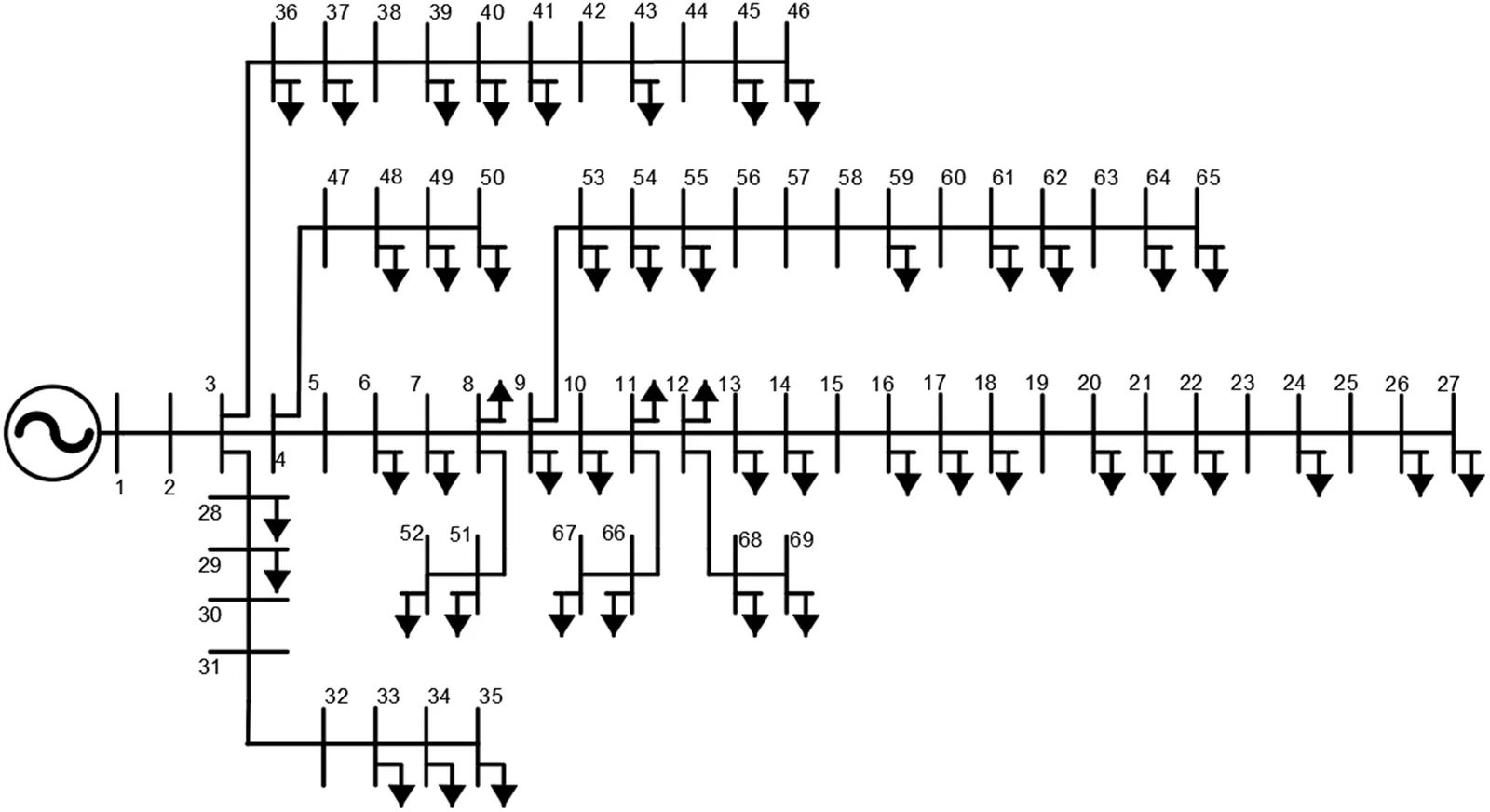
The IEEE 69-bus DS.

In this case, WTDGUs and PVDGUs are considered for connection in the IEEE 69-bus DS. The whole data of the system are shown in S2 Table in S1 File, and its configuration is plotted in Fig 4 [20]. The nonlinear loads are also located at buses No. 8, 12, 18, 22, 24, 34, 46, 55, and 65.

**Fig 5:**
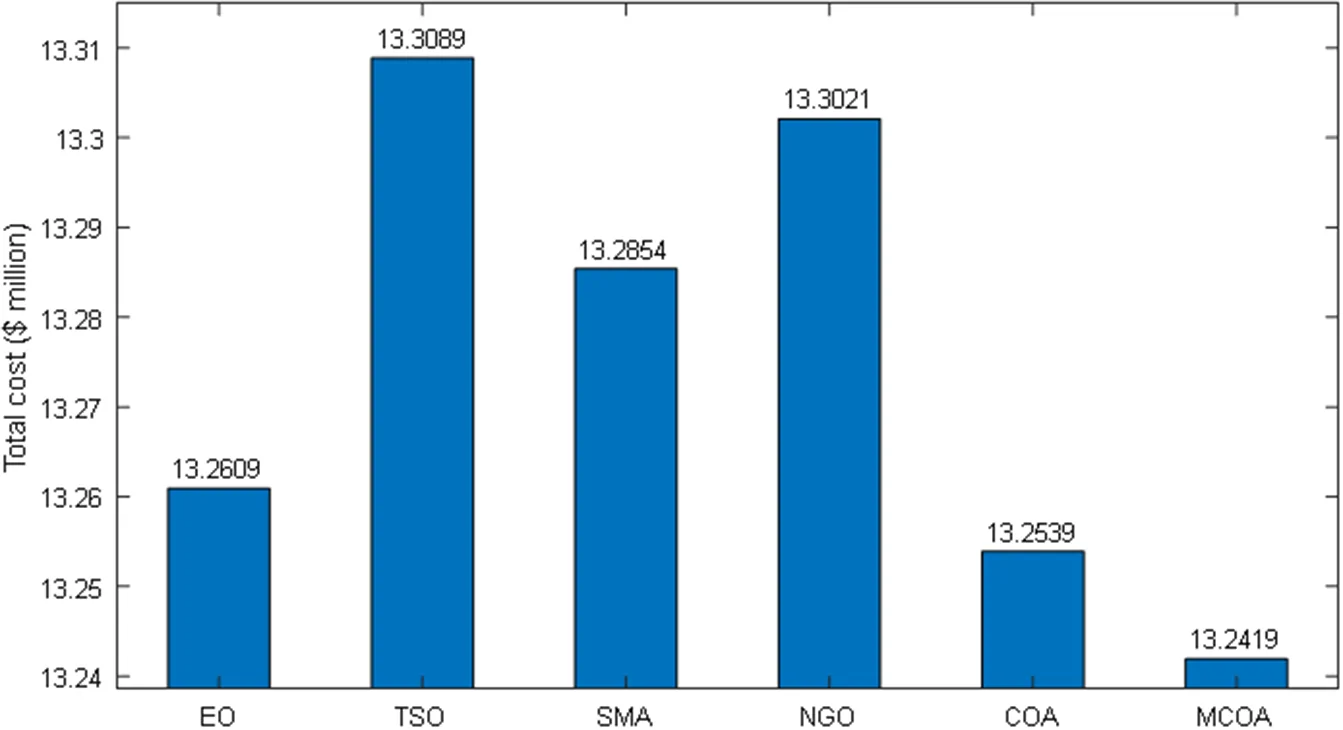
The comparison of the best total cost for operating system in 69-bus system.

Fig 5 presents the best total cost in the trials of the implemented methods, including EO ($13.2609 million), TSO ($13.3089 million), SMA ($13.2854 million), NGO ($13.3021 million), COA ($13.2539 million), and MCOA ($13.2419 million). Clearly, the total cost from the suggested method, MCOA, is the most economical compared to the other methods. The difference in total cost between the suggested method and the compared methods of EO, TSO, SMA, NGO, and COA is up to $19.0 thousand (0.14%), $67.0 thousand (0.51%), $43.5 thousand (0.33%), $60.2 thousand (0.45%), and $12.0 thousand (0.09%), respectively. Although this difference is not large, it still indicates the excellent performance of MCOA compared to the original algorithm and other algorithms. In addition, the economic benefit of applying the optimal solution from the methods compared to the base case is also analyzed in Table 5. In the initial system, without penetration of PVDGUs and WTDGUs in the grid, the total cost includes $17.7384 million from purchasing electricity from the main grid and $412.4749 million from power loss during operation, so the total cost to be paid over 20 years is up to $18.1508 million. However, by using the optimal solution from the implemented methods for placing PVDGUs and WTDGUs in the grid, the total cost is reduced to $13.2609 million, $13.3089 million, $13.2854 million, $13.3021 million, $13.2539 million, $13.2419 million for EO, TSO, SMA, NGO, COA, and MCOA, respectively. Obviously, the solution from MCOA is the most economical, with a cost saving of up to $4.9090 million (equivalent to 27.05%) compared to the base case. Meanwhile, the other solutions only save 26.94% (EO), 26.67% (TSO), 26.81% (SMA), 26.71% (NGO), and 26.98% (COA). In other words, the solution found by the suggested method is better than other methods in addressing the problem of integration of PVDGUs and WTDGUs in the distribution grids.

**Table 5:** The comparison of cost components in the total cost of implemented methods in 69-bus system.

Method	Base system	EO	TSO	SMA	NGO	COA	MCOA
Cost of the investment, OM (${TC}_{DG}^{Inv\_OM},\;$$ million)	0	6.6092	6.5006	6.5797	6.5354	6.6079	6.6095
Cost of purchasing energy from the main grid (${TC}_{DG}^{grid},\;$$ million)	17.7384	6.5603	6.7297	6.6064	6.6755	6.5622	6.5597
Cost of energy loss (${TC}_{DG}^{loss},\;$$ thousand)	412.4749	91.4831	78.5694	99.3829	91.2897	83.8065	72.6193
Total cost (${TC}_{DG},\;$$ million)	18.1508	13.2609	13.3089	13.2854	13.3021	13.2539	13.2419
Saving cost ($ million) =$TC-{TC}_{DG}$	–	4.8899	4.8419	4.8654	4.8487	4.8969	4.9090
Saving cost (%)	–	26.94	26.67	26.81	26.71	26.98	27.05

All the costs considered in this study are also presented in detail in Table 5. The costs (${TC}_{DG}^{Inv\_OM}$ and ${TC}_{DG}^{grid}$) for EO, TSO, SMA, COA and MCOA are ($6.6092 million and $6.5603 million), ($6.5006 million and $6.7297 million), ($6.5797 million and $6.6064 million), ($6.5354 million and $6.6755 million), ($6.6079 million and $6.5622 million) and ($6.6095 million and $6.5597 million), respectively. These numerical results show that the optimal solution from MCOA has higher investment and OM costs but lower electricity import costs from the main grid compared to other methods. Besides, the cost of ${TC}_{DG}^{loss}$ from MCOA is $72.6193 thousand, which is also lower than the remaining methods, such as EO of $91.4831 thousand, TSO of $78.5694 thousand, SMA of $99.3829 thousand, NGO of $91.2897 thousand, and COA of $83.8065 thousand. All these results have proven that the solution from MCOA, although having higher investment and OM, has a more economical electricity purchase cost and power loss cost than all other methods in this case. In summary, since the target of total cost for implementing this project by applying the optimal solution from the suggested method is better than other compared methods, it can be affirmed that MCOA is an effective method in addressing optimization problems in different distribution networks.

The location and the number of installed WTDGUs and PVDGUs by using the solution from applied methods for the two systems are given in S9 Table in S1 File. Selected installation locations by all methods are not the same for both PVDGUs and WTDGUs; however, the total number of wind turbines is the same for all methods with 23 and 24 wind turbines are respectively taken for the first and second systems. Besides, the total power of PVDGUs is not identical, and in fact, the minimum and maximum total power values are 989,400 W and 1,214,400 W for the first system, and 1,087,125 W and 1,205,500 W for the second system. The hourly output power of WTDGUs and PVDGUs is also plotted in S1 and S2 Figures in S1 File, respectively.

#### 5.1.3. The Contributions of WT-PVDGUs to distribution systems.

**Fig 6:**
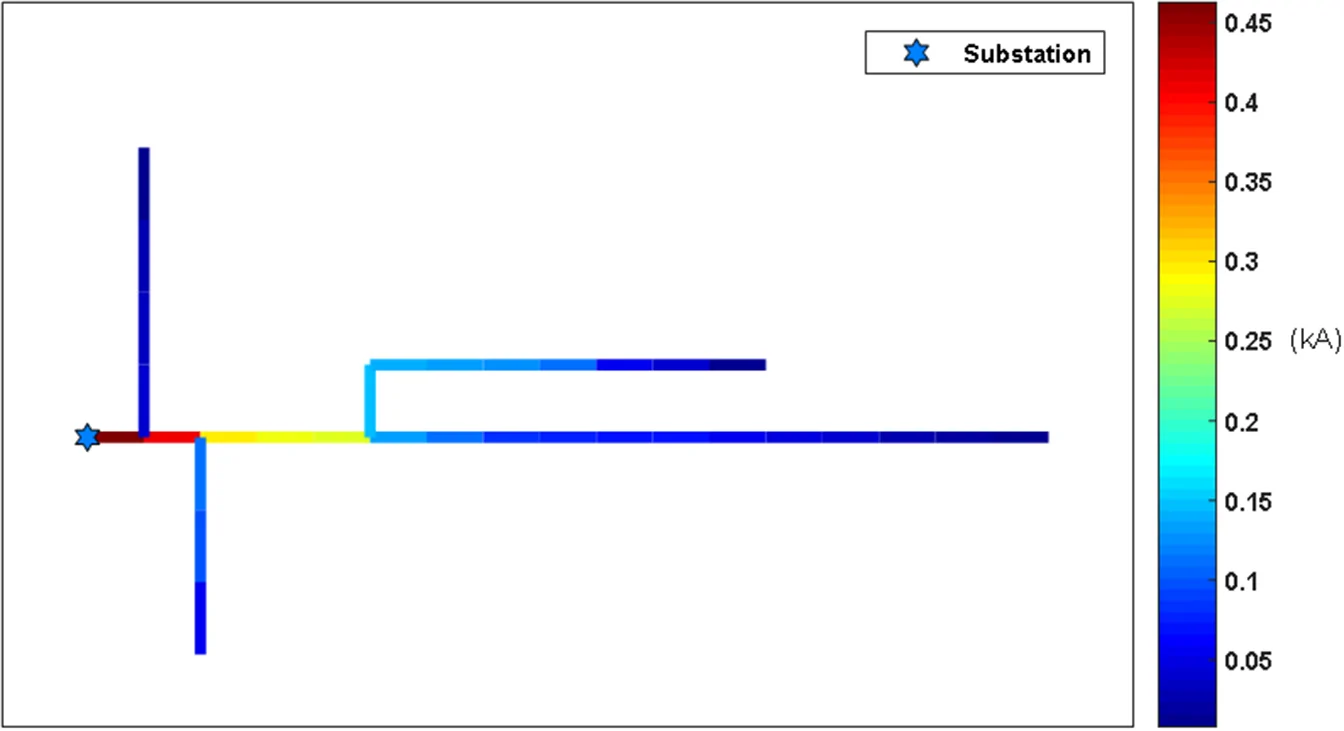
The highest line current (in pu) during the considering period without connecting PVDGUs and WTDGUs in IEEE 33-bus system.

**Fig 7:**
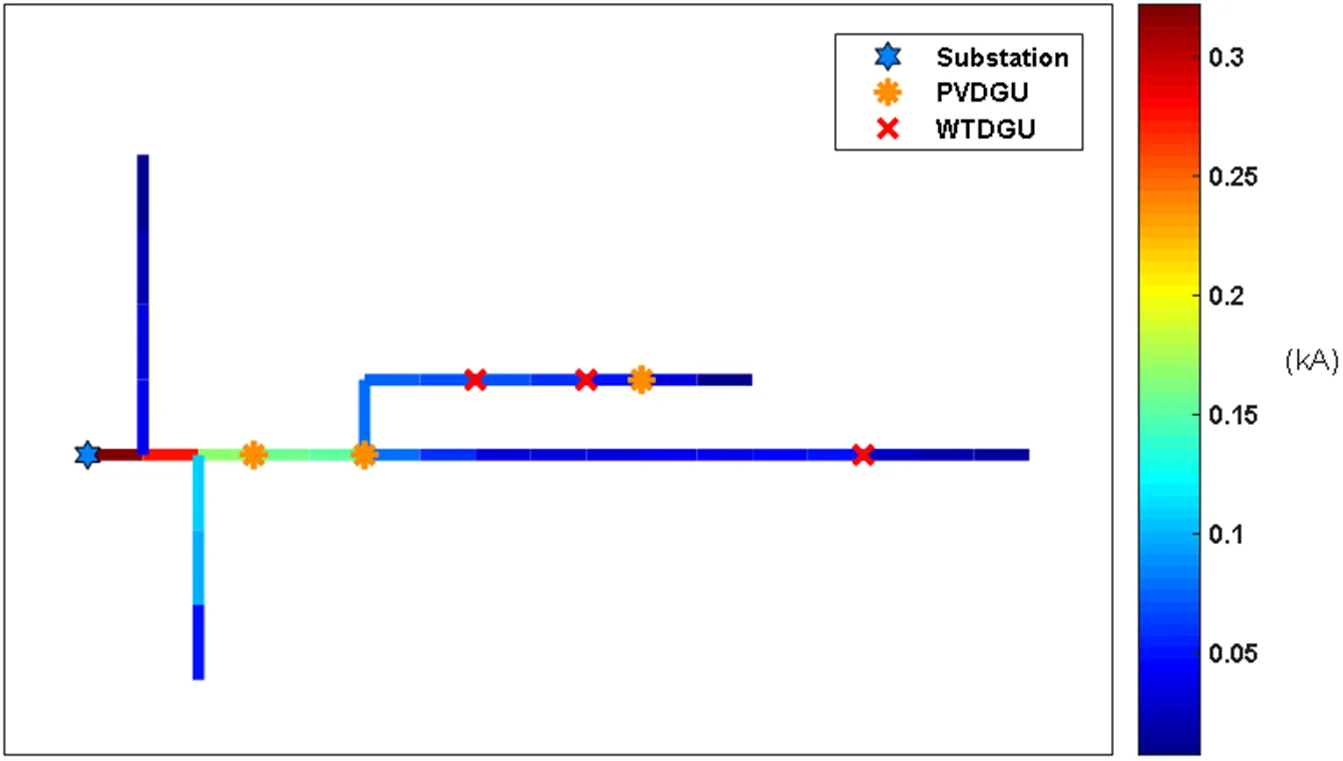
The highest line current (in pu) during the considering period with connecting PVDGUs and WTDGUs in IEEE 33-bus system.

As proven in the two sections above, the connection of WT-PVDGUs in two test systems could bring economic benefits effectively. Thus, this section expresses advantages derived from the installation of WT-PVDGUs in distribution systems from a technical aspect. Specifically, Fig 6 and Fig 7 show the highest current of distribution lines during the considered period in the geographic visualization of IEEE 33-bus DS before and after placing WT-PVDGUs. The two figures are the same in that the current of the base system is much higher than that of the integrated system. For instance, the peak current of line 01 in the base IEEE 33-bus DS is 0.4624 pu, while that is 0.3217 pu in the integrated IEEE 33-bus DS. This has proven to be one of the benefits of installing WT-PVDGUs in reducing the branch current in the power system.

**Fig 8:**
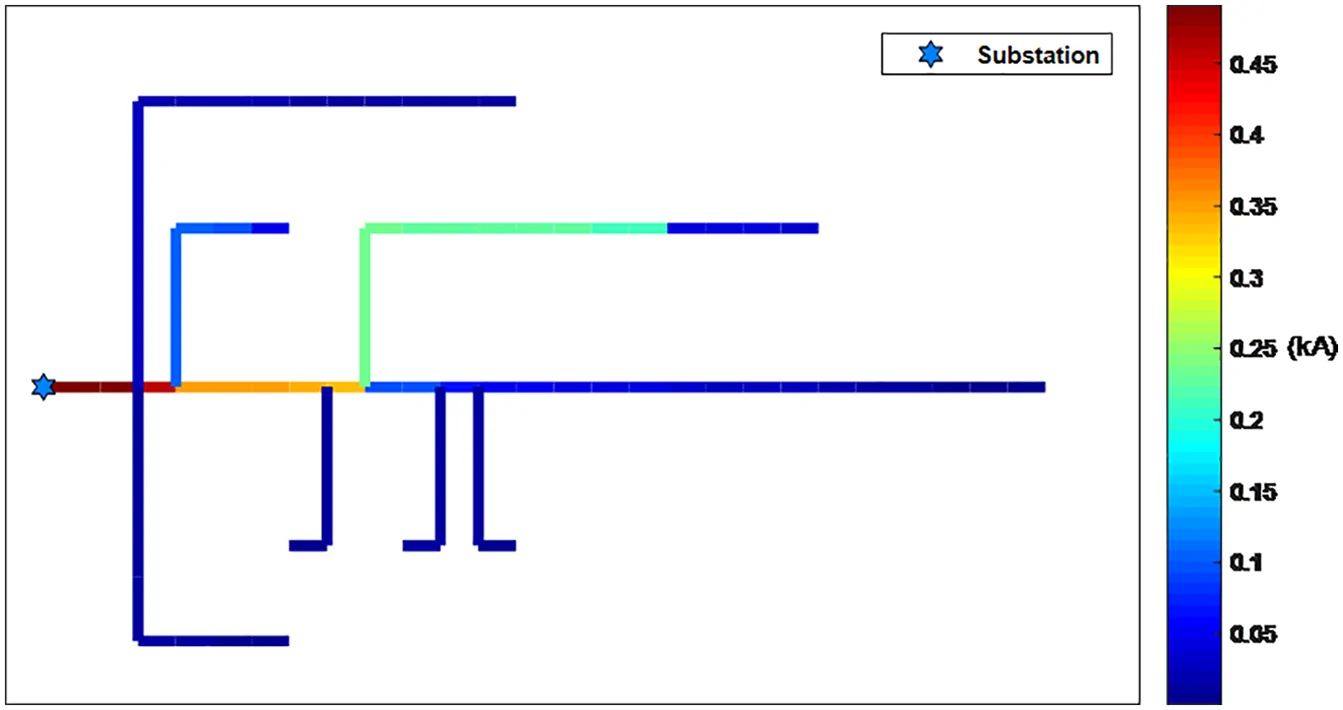
The highest line current (in pu) during the considering period without connecting PVDGUs and WTDGUs in IEEE 69-bus system.

**Fig 9:**
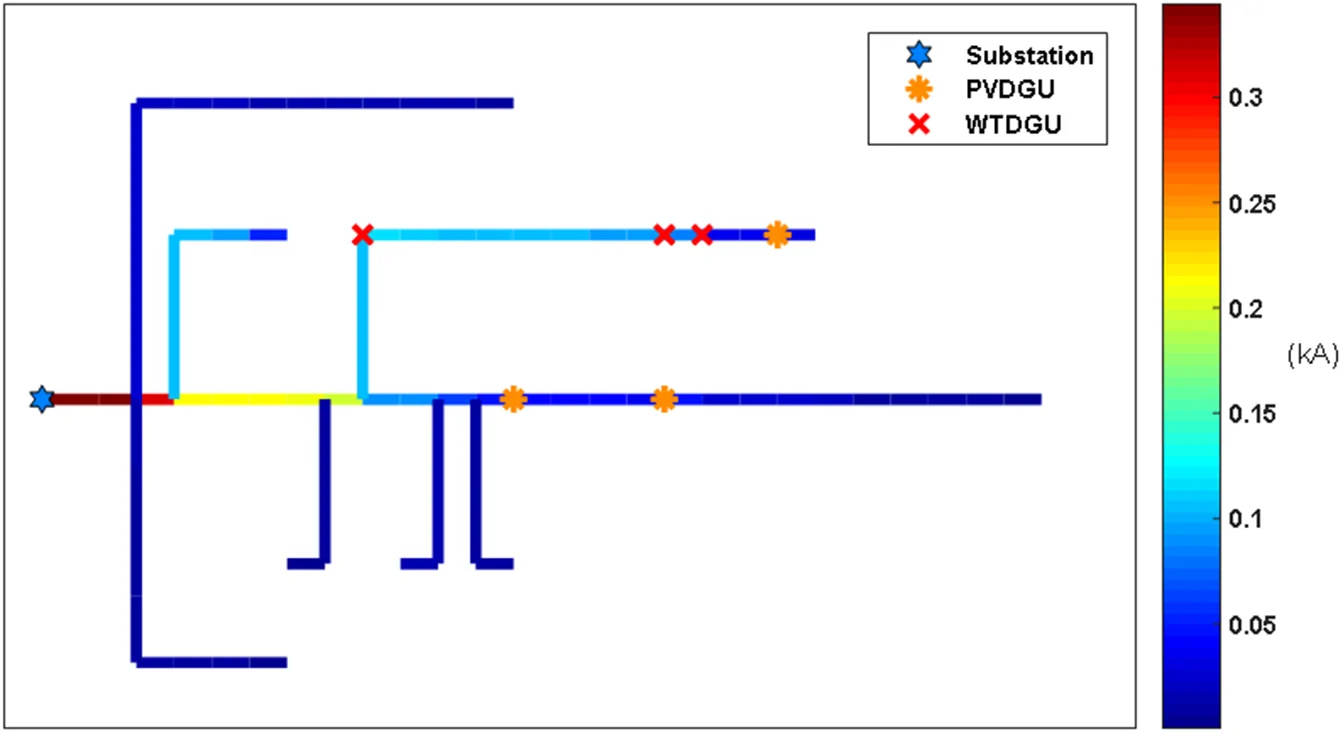
The highest line current (in pu) during the considering period with connecting PVDGUs and WTDGUs in IEEE 69-bus system.

Similarly, the highest current of distribution lines during the considered period in the IEEE 69-bus DS before and after placing WT-PVDGUs is also presented in the geographic visualization as Fig 8 and Fig 9, respectively. The peak current of Line 01 in the base IEEE 69-bus DS is 0.4902 pu while that is 0.3441 pu in the integrated IEEE 69-bus DS. In this regard, the advantage of the WT-PVDGUs is to reduce the current on branches, and this can mitigate transmission power congestion as well as save cost for changing the grid configuration in case of increased load demand due to economic development.

**Fig 10:**
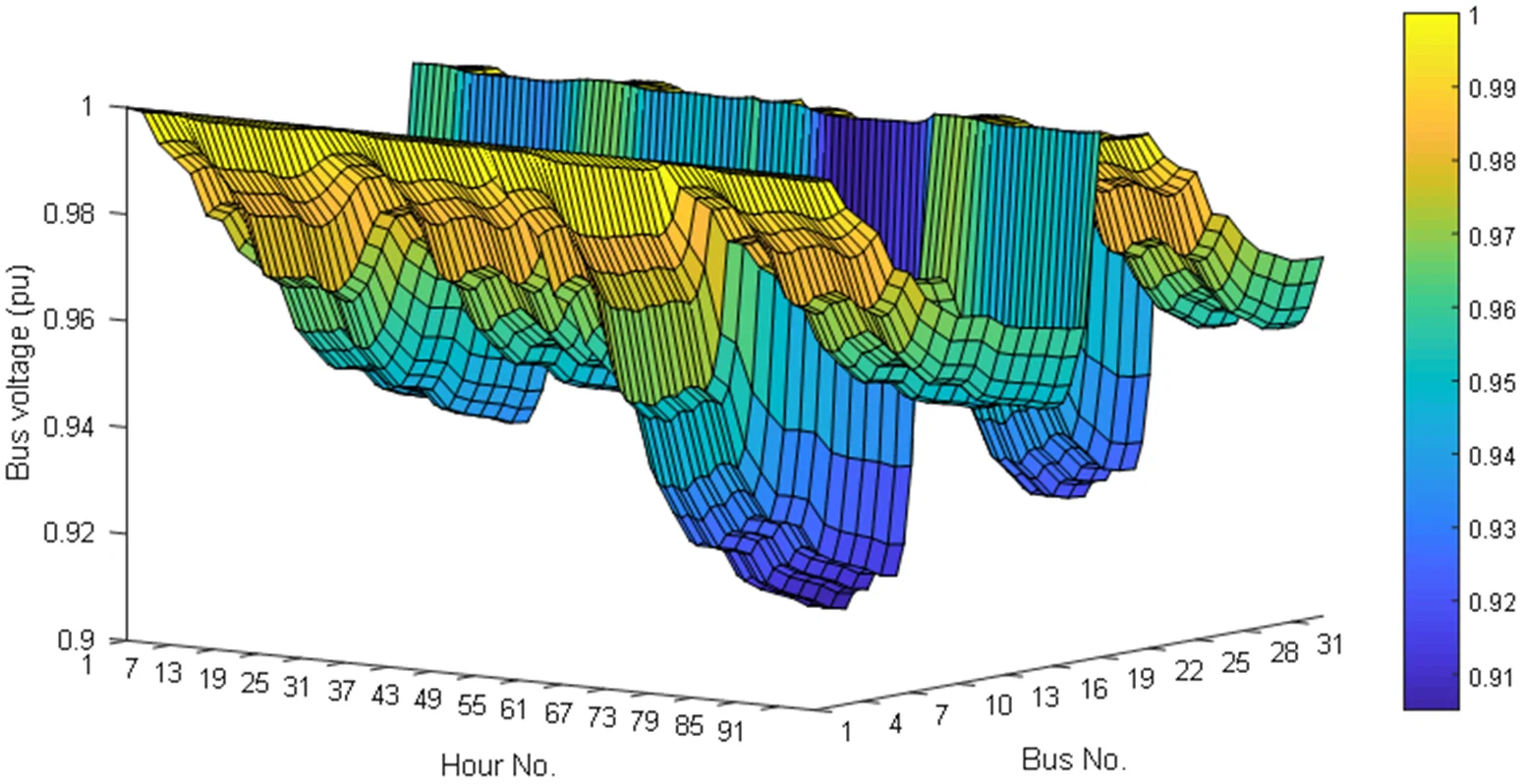
The bus voltage (in pu) before connecting PVDGUs and WTDGUs in IEEE 33-bus system.

**Fig 11:**
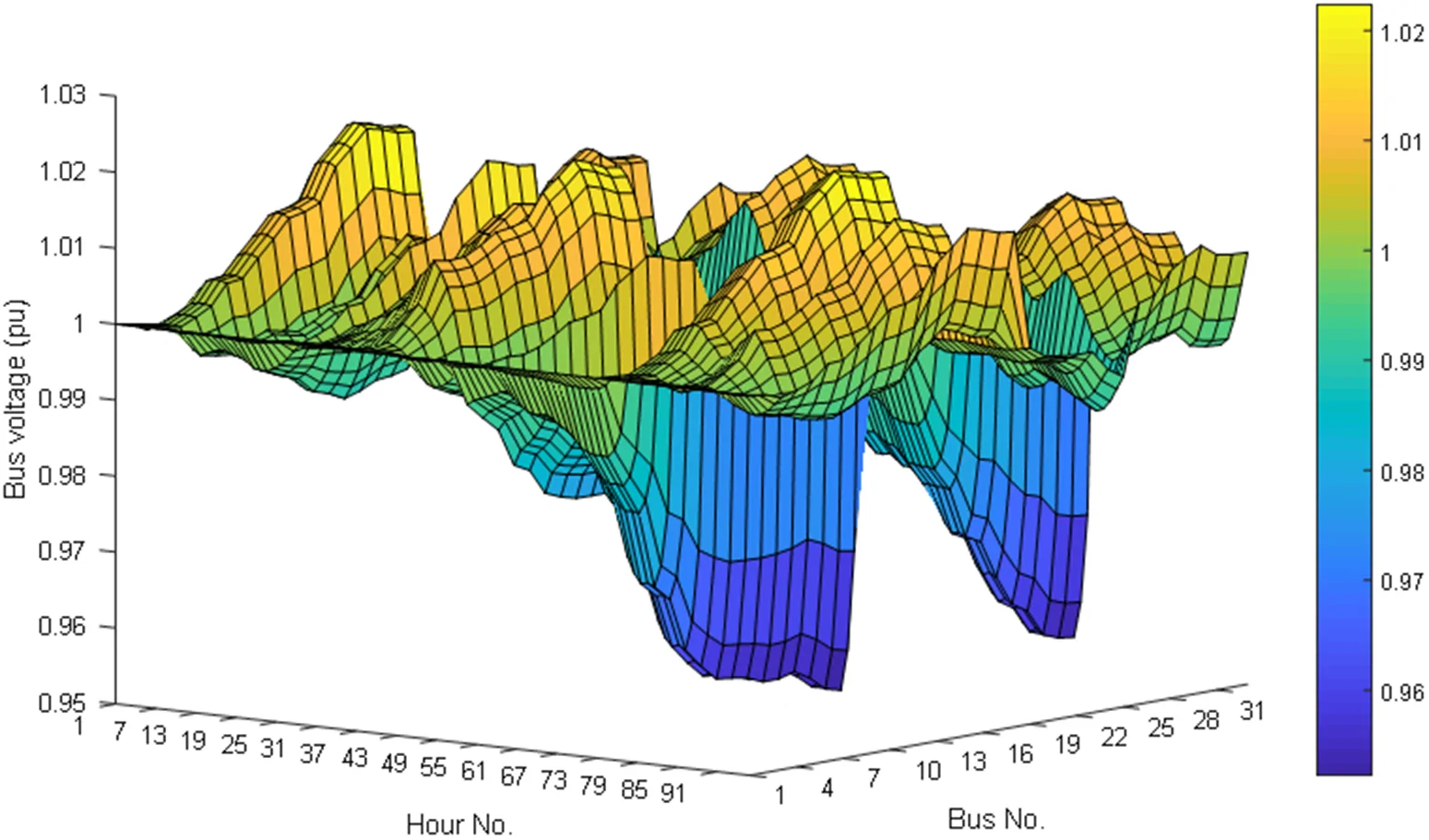
The bus voltage (in pu) after connecting PVDGUs and WTDGUs in IEEE 33-bus system.

**Fig 12:**
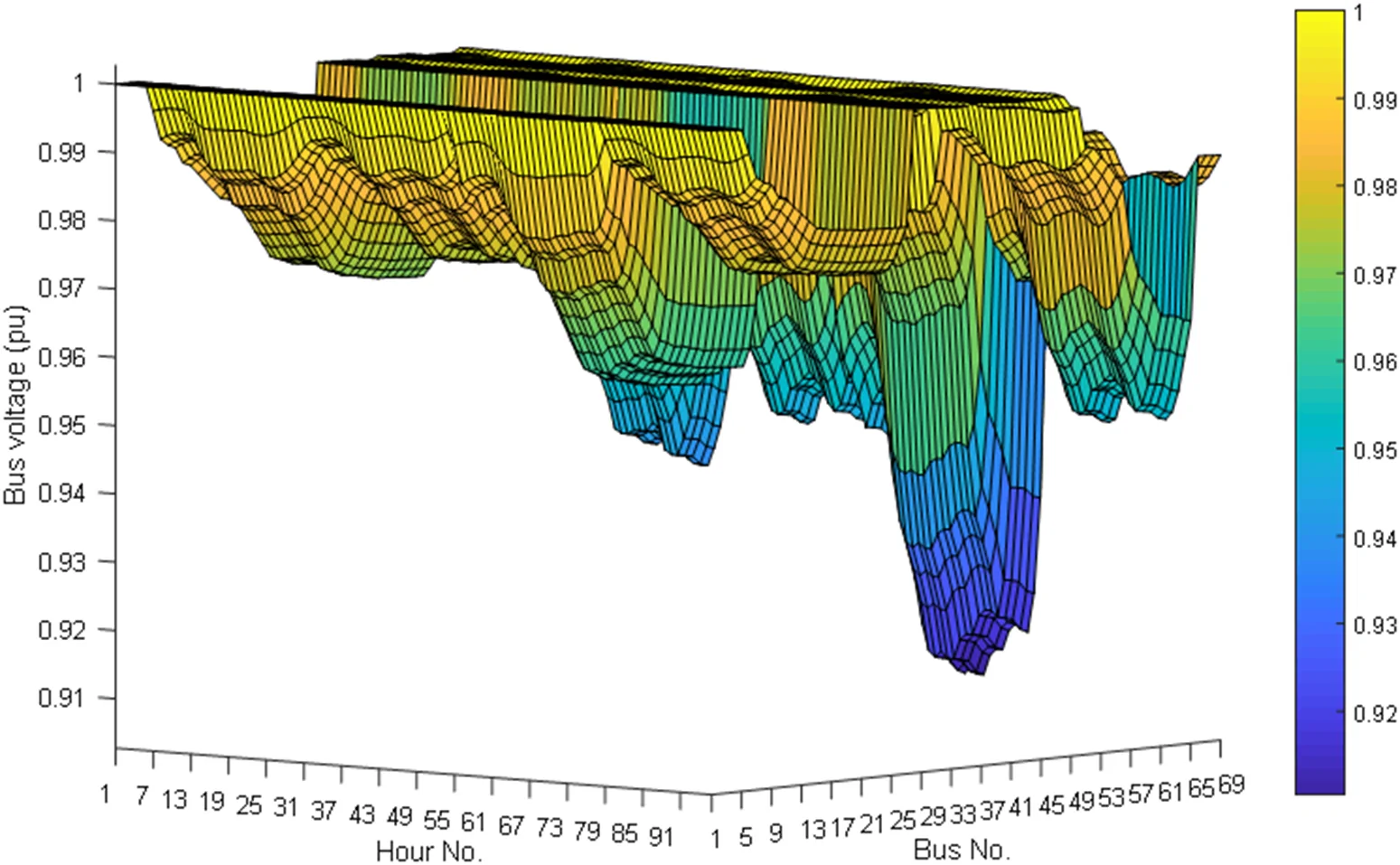
The bus voltage (in pu) before connecting PVDGUs and WTDGUs in IEEE 69-bus system.

**Fig 13:**
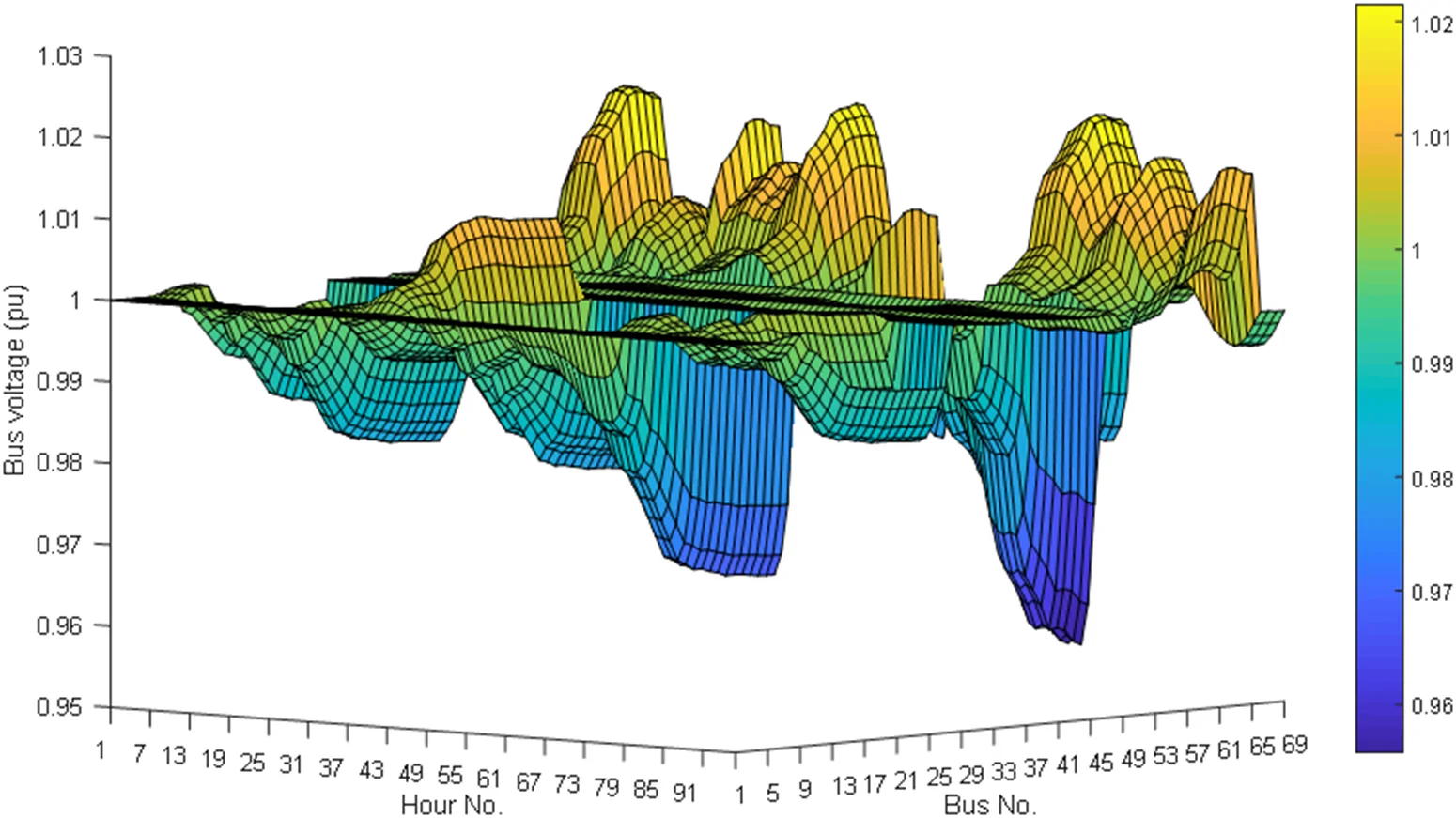
The bus voltage (in pu) after connecting PVDGUs and WTDGUs in IEEE 69-bus system.

Furthermore, Figs 10–13 also presents the voltage profile of the whole system over four seasons. Specifically, the lowest voltage is increased from 0.9051 pu to 0.9523 pu for the IEEE 33-bus DS as plotted in Figs 10 & 11, and from 0.9105 pu to 0.9558 pu for IEEE 69-bus DS as plotted in Figs 12 & 13, based on the penetration of WT-PVDGUs. Overall, during the considering period, the voltage of buses only fluctuates in the range of [0.9523, 1.0223] (pu) in the first system and in the range of [0.9558, 1.0215] (pu) in the second system. Obviously, the two integrated systems fully satisfied the given constraints of [0.95, 1.05] (pu), and this has also contributed to demonstrating the technical benefit of voltage improvement due to the penetration of generation sources into the power grids.

**Fig 14:**
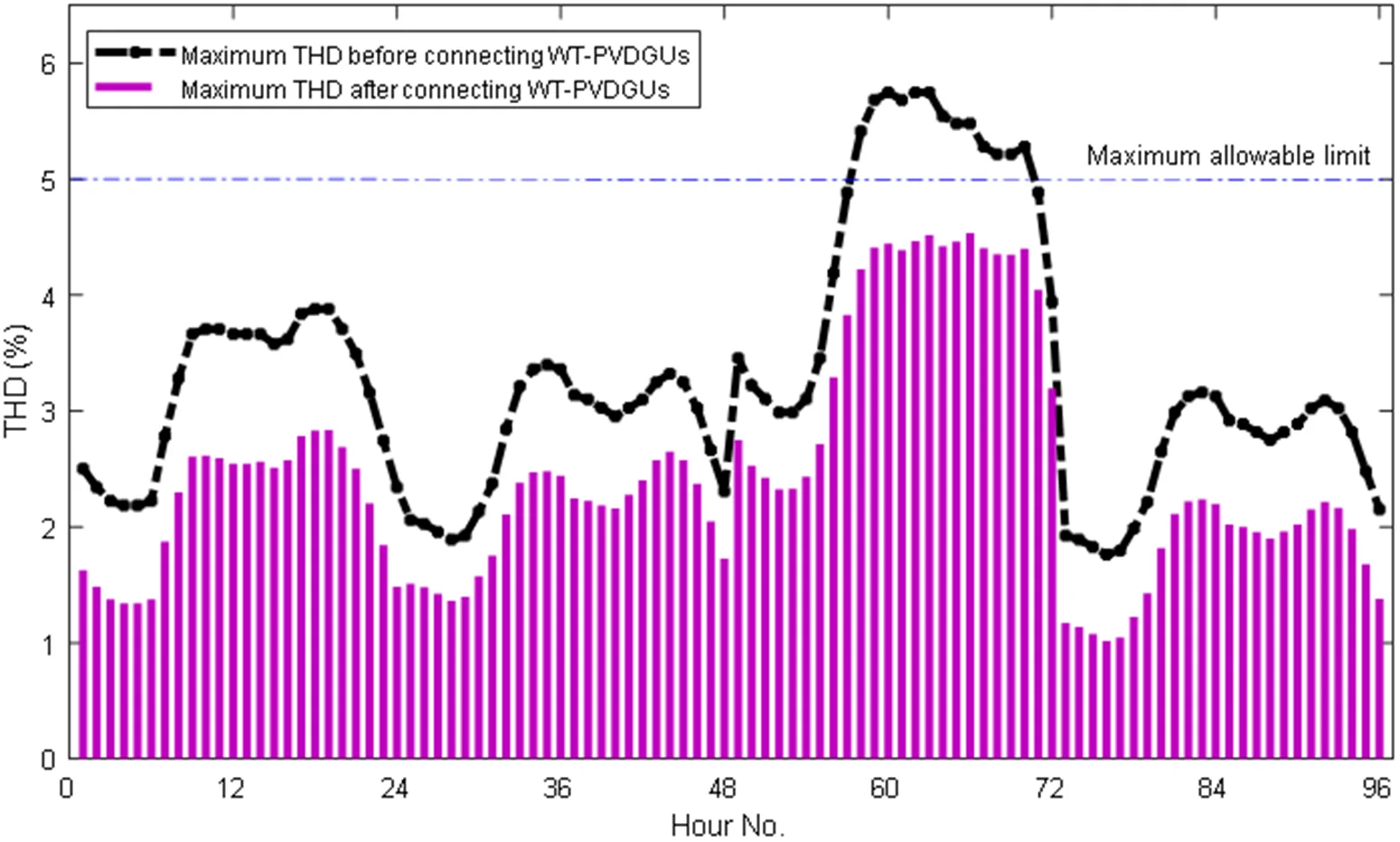
Maximum THD values without and with connecting PVDGUs and WTDGUs in IEEE 33-bus system.

**Fig 15:**
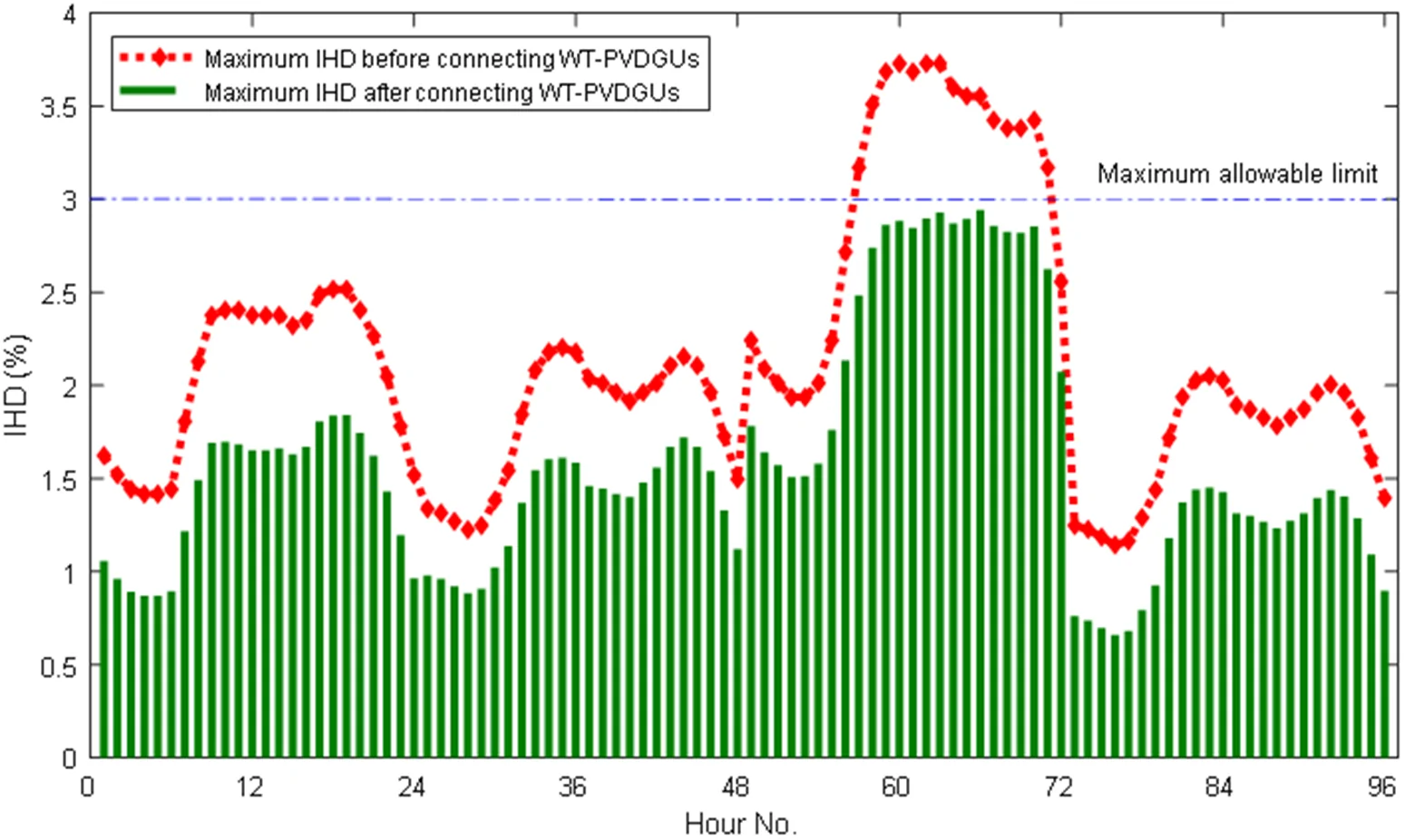
Maximum IHD values without and with connecting PVDGUs and WTDGUs in IEEE 33-bus system.

**Fig 16:**
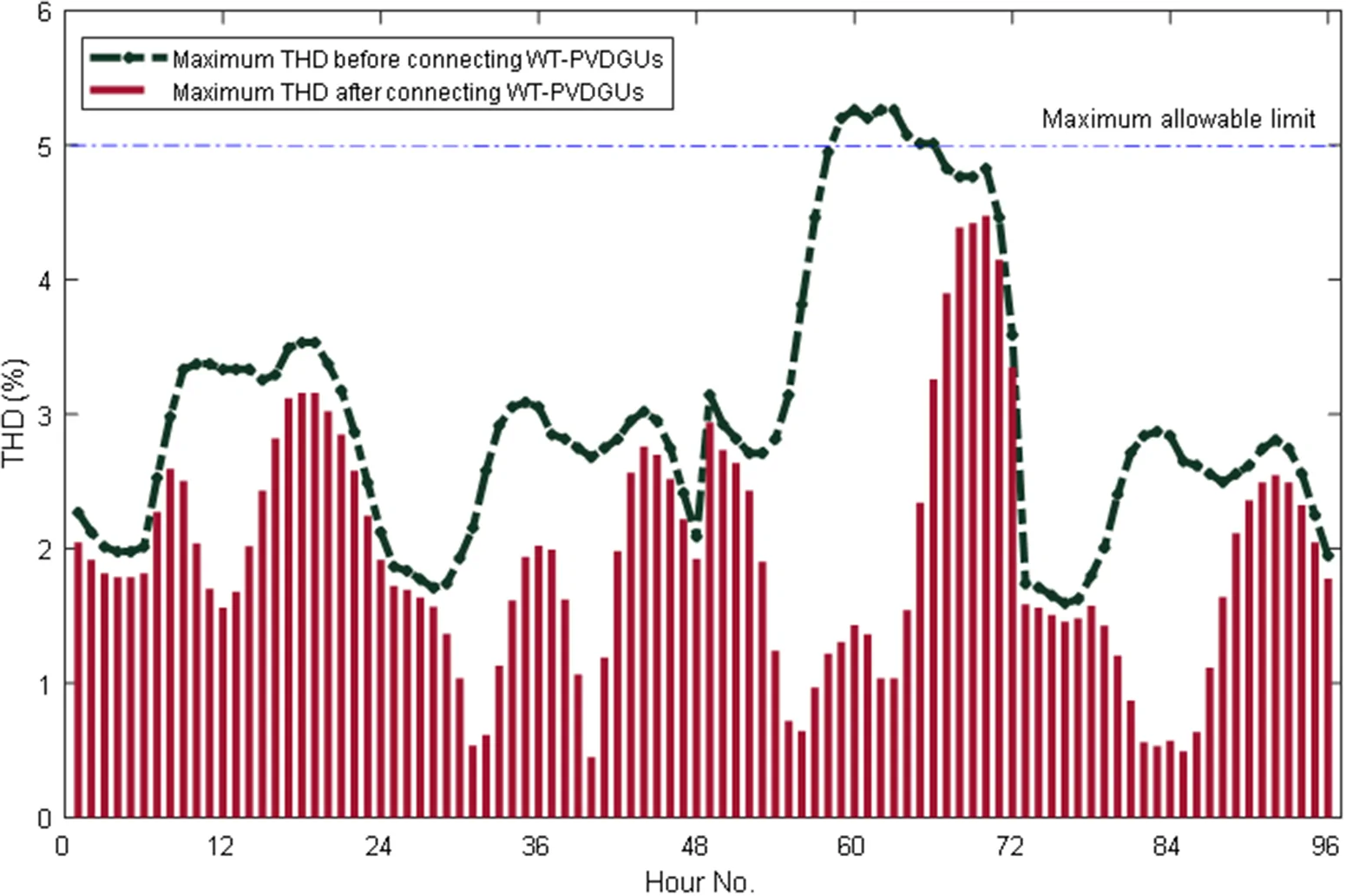
Maximum THD values without and with connecting PVDGUs and WTDGUs in IEEE 69-bus system.

**Fig 17:**
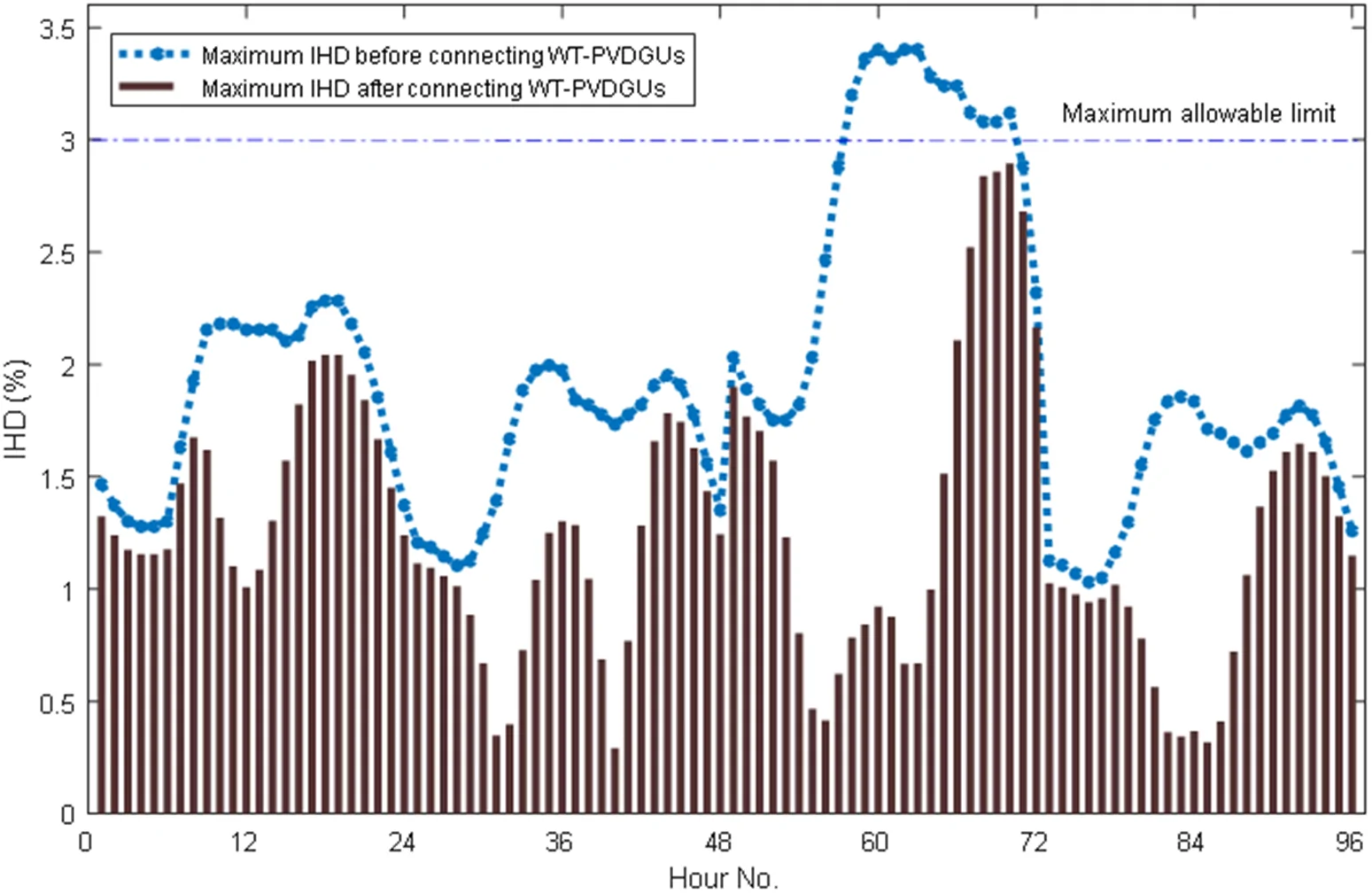
Maximum IHD values without and with connecting PVDGUs and WTDGUs in IEEE 69-bus system.

Additionally, Figs 14–17 also show the maximum values of total and individual harmonic distortion (THD and IHD) before and after connecting WT-PVDGUs in the two test systems. The view during the considering period sees that base systems always have much higher values of THD and IHD than the integrated systems with WTDGUs and PVDGUs, even though these base systems violated the standard of IEEE 519. In detail, these values are 5.7491% and 3.7295% in IEEE 33-bus DS, and 5.2646% and 3.4025% in IEEE 69-bus DS for THD and IHD, respectively, at the base case as shown in Figs 14 & 15. However, in the integrated systems, they always satisfy the standard of IEEE 519 with specific values of 4.2180% and 2.7365% in the first system, and 4.4783% and 2.8943% in the remaining system, as shown in Figs 16 & 17. In other words, thanks to the reasonable penetration of WTDGUs and PVDGUs, the harmonic indices such as THD and IHD are significantly reduced and satisfy the standard of IEEE 519 of 5% (for THD) và 3% (for IHD) as the established constraints. This is also considered an additional benefit from the application of the optimal solution regarding the determination of the installation of distributed sources in the distribution grids.

#### 5.1.4. Computation speed from applied algorithms.

The optimal solution for integrating wind turbine and photovoltaic distributed generation units is successfully determined by the implemented methods, as shown in S7 Table in S1 File. However, to ensure a fair and objective evaluation, the initial setup parameters and the data processing speed in loops for each method are also compared.

Specifically, the number of iterations and populations are surveyed, respectively, within the range from 130 to 170 (the step size is 20) and from 16 to 24 (the step size is 4) for applied methods in the test cases, and the appropriate tuning values are clearly reported in Table 6. In the first test system, EO, TSO, SMA, and NGO are configured with an iteration limit of 150 and a population size of 20, while those of COA and MCOA are 150 and 16. For the second system, the loop setup is adjusted by increasing the iteration count to 170 for all methods, while the population settings remained similar to the first system to ensure the best convergence of methods. As mentioned, MCOA is an improved version of COA with more efficient solution generation equations; therefore, MCOA has inherited the outstanding features of the original algorithm, and they have the same characteristic in producing a solution number for each iteration. In detail, these two algorithms divided the population into four groups with four members in each group, and they produced four more new solutions in addition to their population. In brief, all methods have the same number of evaluations for one run, and the settings are fair for all algorithms. However, due to the unique characteristics, the mean computation time for each method is different. For the first system, it takes MCOA about 0.8345 hours for each trial, while it takes others a longer time, from 0.8479 to 1.7011 hours for COA, TSO and NGO. For the second system, the time spent by MCOA is about 1.6156 hours for each trial, while it is from 1.6329 hours to 3.3528 hours for COA, TSO and NGO. Obviously, it can be seen that for a larger system, a greater number of iterations and longer simulation time are required to achieve the optimal solution. Additionally, in both cases, the computation time has not improved significantly, but it has also helped demonstrate the superiority of MCOA over the original method and others. In summary, MCOA is superior to other algorithms in terms of robustness, stability, and search speed.

**Table 6:** Computation speed of implemented methods for IEEE 33- bus and IEEE 69- bus DSs.

Method	IEEE 33- bus DS			IEEE 69- bus DS		
	Population	Iteration number	Average computation time (hours)	Population	Iteration number	Average computation time (hours)
EO	20	150	0.6913	20	170	1.3997
TSO	20	150	1.6665	20	170	2.9750
SMA	20	150	0.6992	20	170	1.4044
NGO	20	150	1.7011	20	170	3.3528
COA	16	150	0.8479	16	170	1.6329
MCOA	16	150	0.8345	16	170	1.6156

### 5.2. Case 2: Considering the fixed output of DGUs

To evaluate effectiveness of the introduced method in the research trends of this field, this study implemented an additional simulation considering the fixed output of DGUs at the peak load level, with the population as reported in Table 6, and the number of iterations are selected as 100 and 140 from the survey for IEEE 33- bus and IEEE 69- bus DSs, respectively. In this case, the optimal results are compared with recently published studies (from 2017 to 2025) in both first and second systems, with the main objective of minimizing total power loss from the penetration of three DGUs. The achieved result is compared to other published methods as presented in Table 7.

**Table 7:** The comparison results of compared methods and suggested method.

IEEE 33- bus DS				
Ref.	Online	Applied method	Optimal location and sizing of DGs (Bus/ Capacity in MW)	Reduction in power losses
[67]	2017	MOTA	7/ 0.980; 14/ 0.960; 30/1.340	52.40%
[39]	2017	HGWO	13/ 0.802; 24/ 1.090; 30/ 1.054	64.40%
[68]	2018	EMA	30/ 0.9766; 24/ 1.1691; 12/ 0.9435	64.32%
[69]	2018	WCA	14/ 0.8546; 24/ 1.1017; 29/ 1.181	65.00%
[70]	2019	SSA	13/ 0.7536; 23/ 1.1004; 29/ 1.0706	64.80%
[71]	2020	CSCA	13/ 0.871; 24/ 1.0915; 30/ 0.9541	64.50%
[72]	2021	CMSFS3	13/ 0.802; 30/ 1.054; 24/ 1.091	65.50%
[73]	2023	WOA	24/ 1.0913; 13/ 0.8017, 30/ 1.0536	65.50%
[74]	2024	WSA	30/ 1.0475; 24/ 1.0938; 14/ 0.7344	65.45%
[75]	2025	NSGA-II	24/ 1.069; 14/ 0.828; 30/ 1.194	63.88%
	2026	MCOA (Introduced)	14/ 0.7708; 24/ 1.0969; 30/ 1.0658	65.51%
**IEEE 69- bus DS**				
**Ref.**	**Online**	**Applied method**	**Optimal location and sizing of DGs (Bus/ Capacity in MW)**	**Reduction in power losses**
[39]	2017	HGWO	11/ 0.527; 17/ 0.380; 61/1.718	69.14%
[69]	2018	WCA	61/ 0.775; 62/ 1.105; 23/ 0.438	68.20%
[70]	2019	SSA	17/ 0.380; 10/ 0.527; 60/ 1.718	69.10%
[71]	2020	CSCA	17/ 0.3660; 61/ 1.6759; 67/ 0.6525	68.85%
[72]	2021	CMSFS3	11/ 0.527; 61/ 1.719; 18/ 0.380	69.07%
[76]	2022	TSO	9/ 0.8251; 22/ 0.4051; 61/1.6501	68.77%
[73]	2023	WOA	66/ 0.4598; 18/ 0.3996; 61/ 1.7270	68.96%
[74]	2024	WSA	21/ 0.3463; 61/ 1.6919; 67/ 0.4451	69.00%
[75]	2025	NSGA-II	17/ 0.5678; 64/ 0.3748; 61/ 1.4977	68.12%
	2026	MCOA (Introduced)	18/ 0.3710; 61/ 1.7189; 11/ 0.5354	69.17%

**Fig 18:**
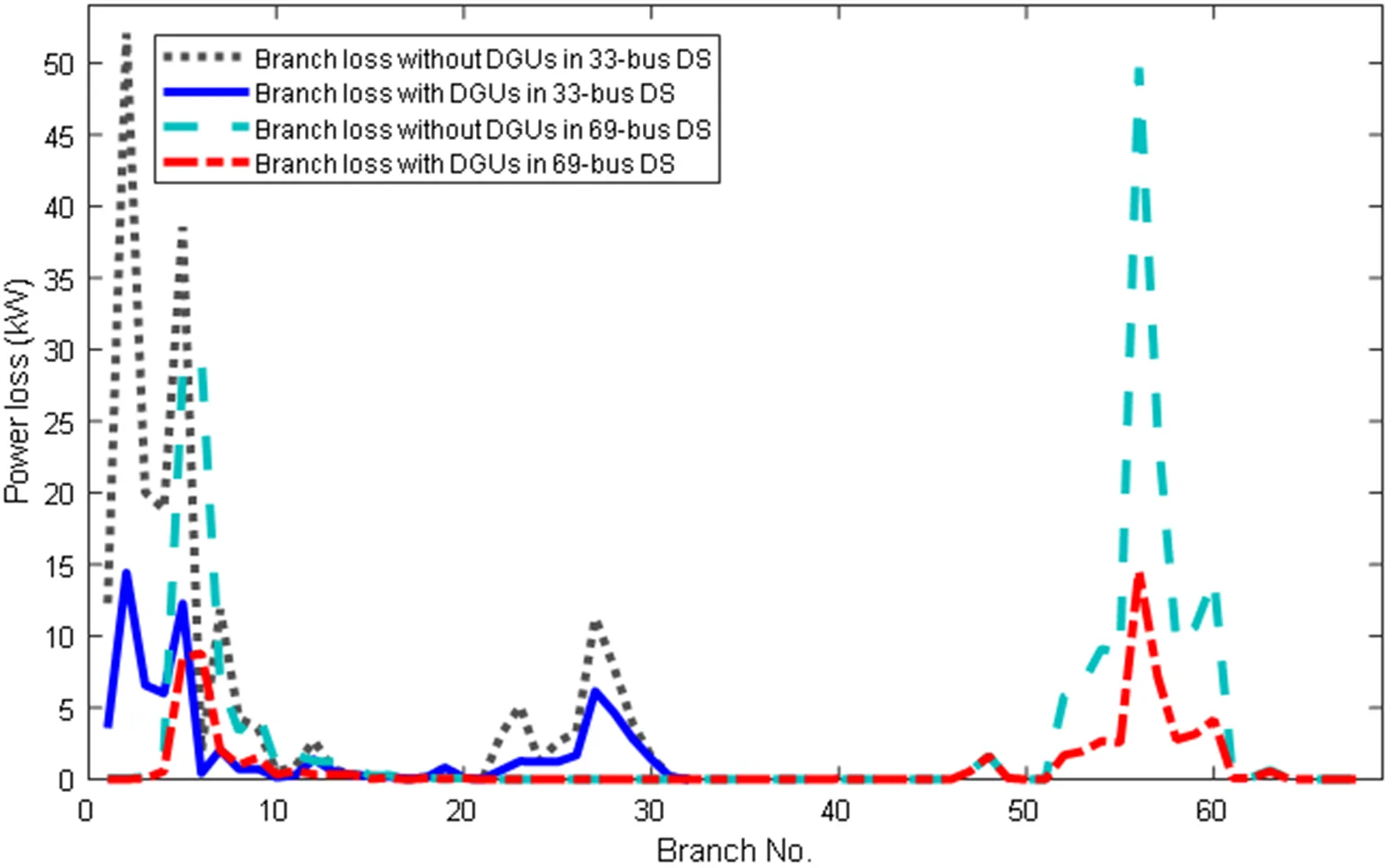
Branch power losses without and with DGUs in both systems.

**Fig 19:**
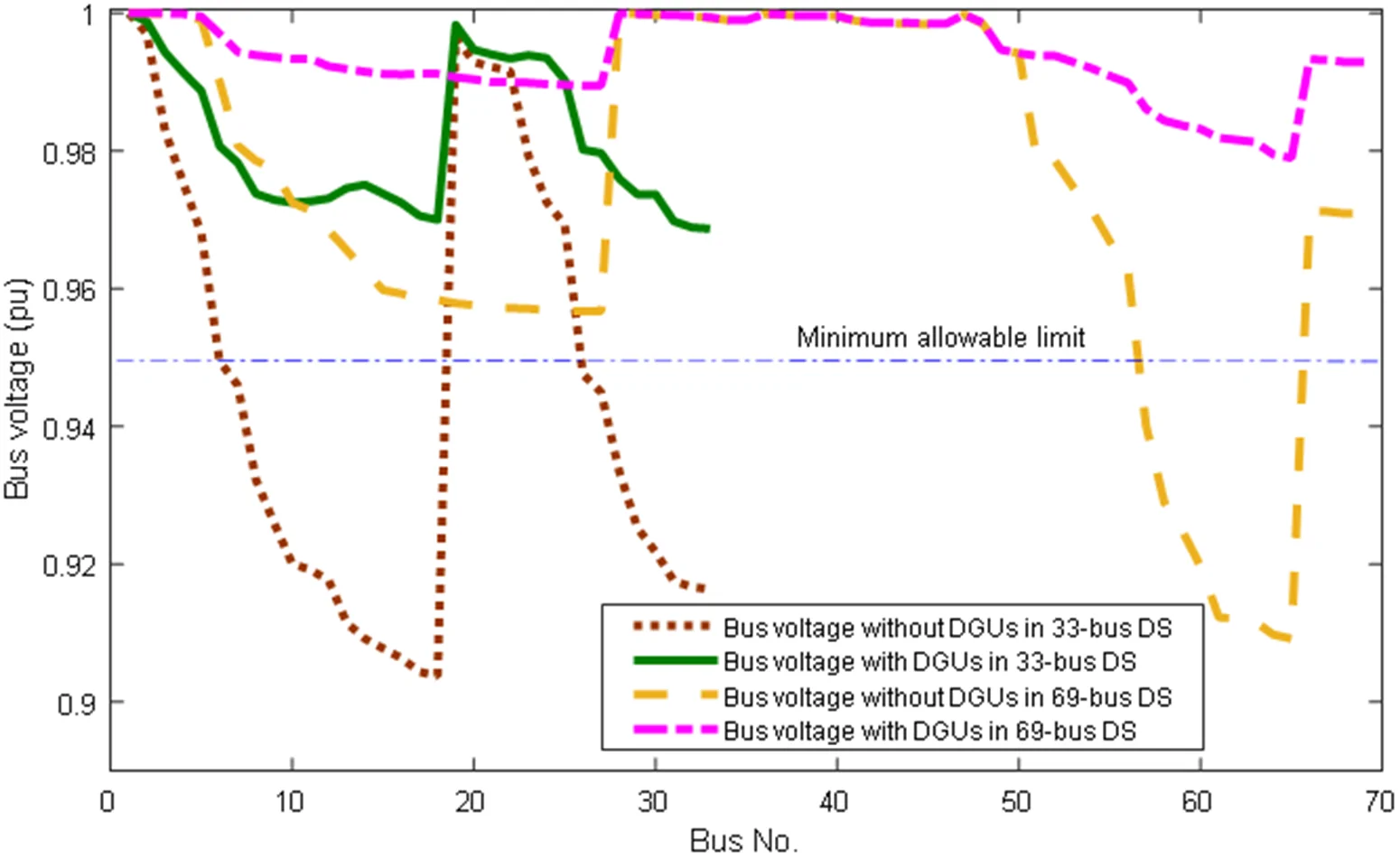
Bus voltage without and with DGUs in both systems.

**Fig 20:**
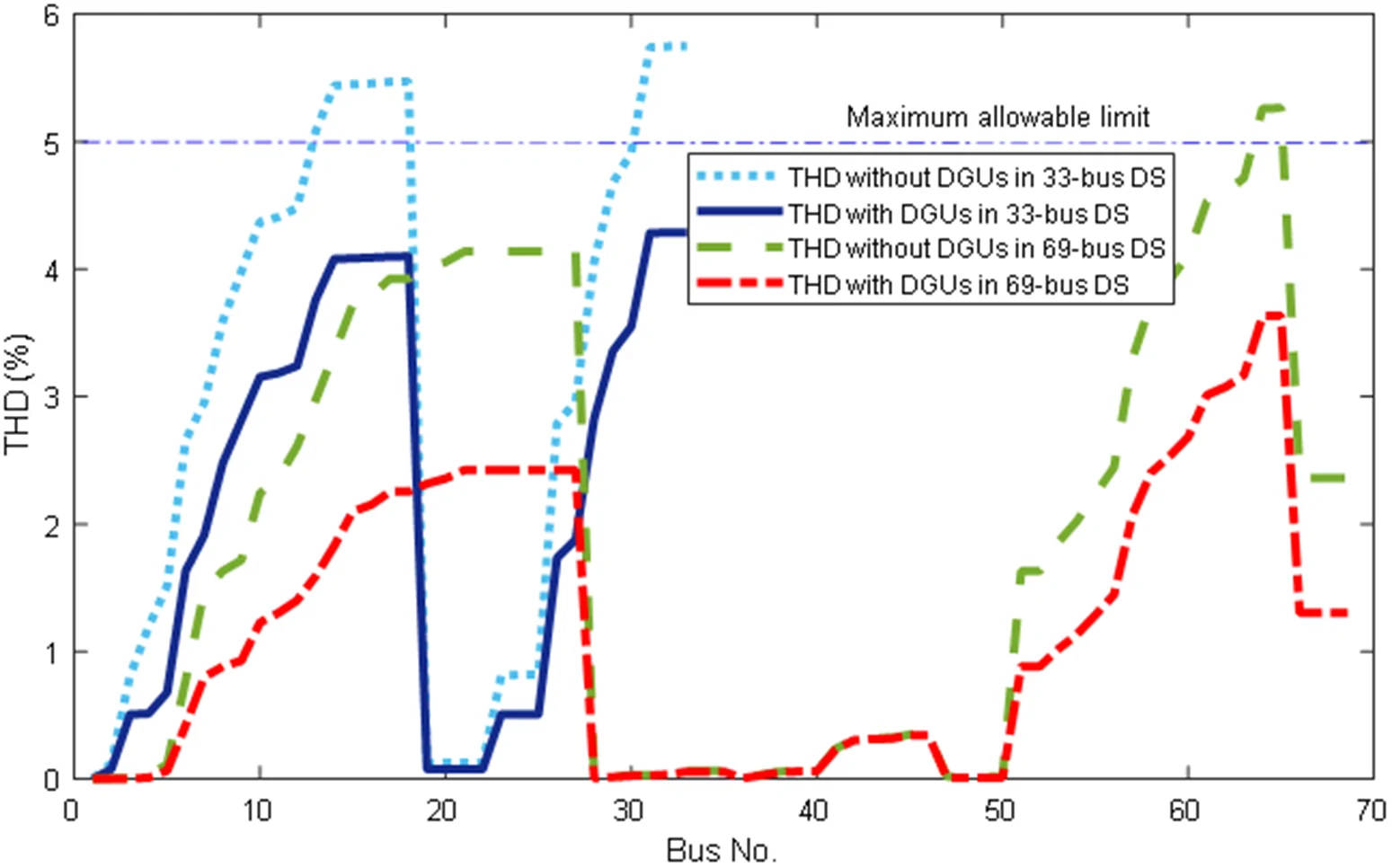
THD values without and with DGUs in both systems.

**Fig 21:**
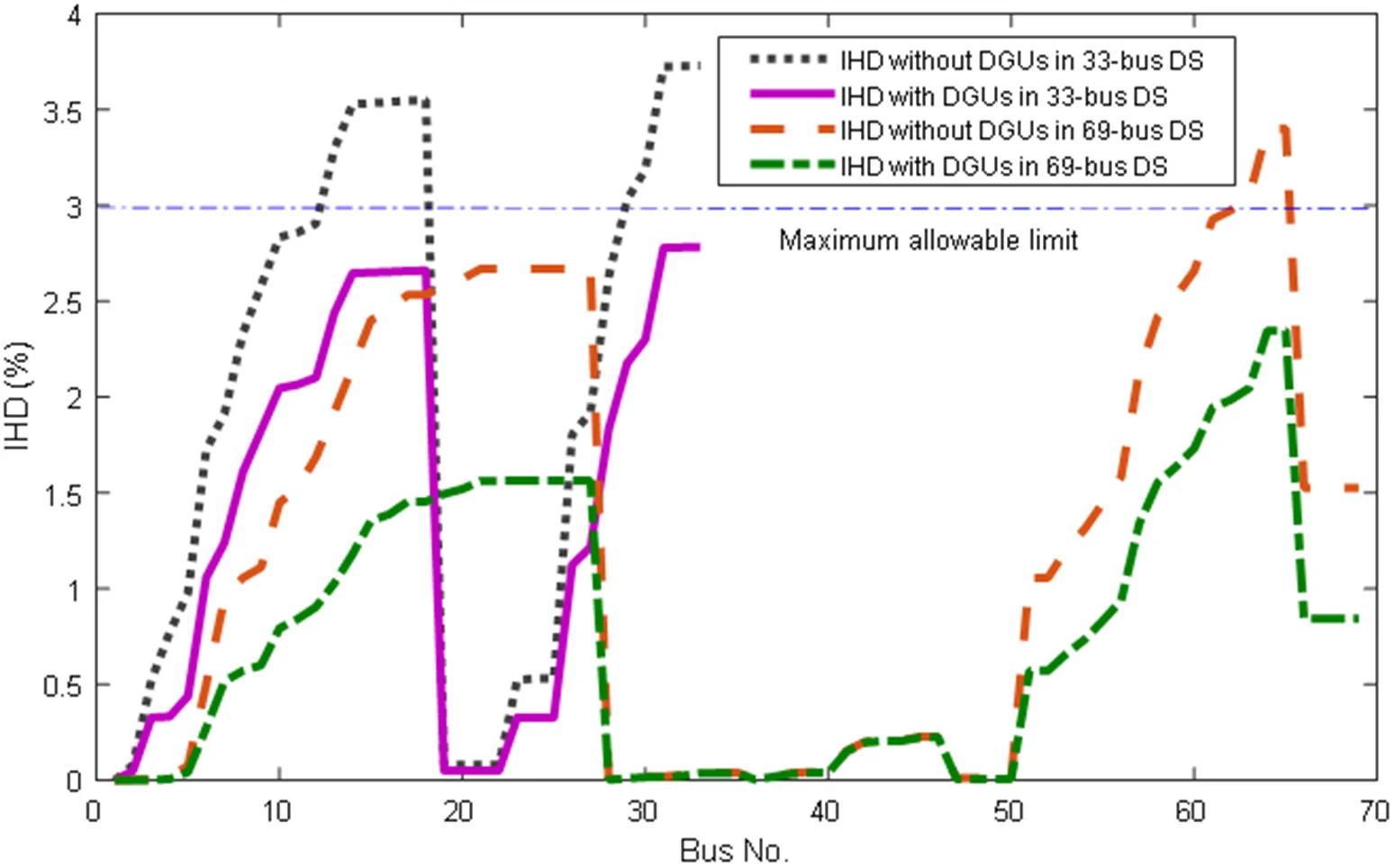
IHD values without and with DGUs in both systems.

Thanks to the optimal solution for connecting DGUs that operate at unity power factor, the total power loss in the 33-bus DS is strongly decreased with a reduction in losses (PLR) of up to 65.51%. However, the determined PLR values from optimal solutions of published methods, such as MOTA [67], HGWO [39], EMA [68], WCA [69], SSA [70], CSCA [71], CMSFS3 [72], WOA [73], WSA [74] and NSGA-II [75], are only varied between 52.4% and 65.50% as presented in Table 7. Obviously, the PLR value of MCOA is the highest, so it can be declared that MCOA is the best method compared to other methods in this case. Similarly, the optimal result from MCOA is also compared with those of many other methods in the larger-scale IEEE 69-bus DS. As shown in Table 7, the total power loss after the penetration of three DGs is significantly reduced, and its PLR value reached approximately 69.17%. Meanwhile, this value only ranged from 68.12% to 69.14% for nine published methods, including HGWO [39], WCA [69], SSA [70], CSCA [71], CMSFS3 [72], TSO [76], WOA [73], WSA [74] and NSGA-II [75]. This indicates that the suggested method yielded the best result with the highest PLR value, making it a truly robust method for solving the optimization problem compared to recently published methods. As mentioned, with the proper integration of DGUs in the distribution grid, the total losses on the network branches have also been minimized as shown in Fig 18. In detail, total branch losses are decreased from 211.0 kW to 72.7827 kW (the reduction amount of 138.2173 kW) for the first system and from 224.5 kW to 69.2065 kW (the reduction amount of 155.2935 kW) for the second system. This is considered a significant benefit by the DGUs connection. Besides the advantage of cutting losses, the optimal solution for integration of DGUs in power grids from the suggested method also offers benefits in terms of voltage enhancement and harmonic mitigation. Specifically, the weakest voltage in the system is pulled up from 0.9038 (pu) to 0.9687 (pu) and from 0.9092 (pu) to 0.9790 (pu) for the 33- bus DS and the 69- bus DS, respectively, as plotted in Fig 19, and this satisfies the bus voltage constraint of 0.950 (pu) to 1.050 (pu). Moreover, harmonic indices of THD and IHD are also reduced, as shown in Figs 20 and 21. Specifically, the THD and IHD indices in the first system are mitigated from (5.7491% and 3.7295%) to (4.2884% and 2.7826%), and from (5.2647% and 3.4025%) to (3.6346% and 2.3482%) in the second system, respectively. The obtained results for harmonic distortion also meet the IEEE 519 standard of 5.0% for the THD index and 3.0% for the IHD index. Based on these numerical results, it can be affirmed that the suitable integration of DGUs can bring multiple benefits in reducing losses, increasing voltage, and reducing harmonics in distribution power grids.

## 6. Conclusions

In this study, a novel powerful MCOA was applied to solve the optimization problem of integrating wind turbines and photovoltaic distributed generation units in the distribution systems with high impacts of nonlinear loads. The study examined two cases, including the consideration of the uncertainty of DGUs and the consideration of the fixed output of DGUs. In the first case, the main purpose is to reduce the total system costs and also meet the stated technical constraints after placing WT-PVDGUs concerning the wind speed and solar irradiance uncertainties. Weibull and Beta pdfs were also used to estimate the hourly wind speed and solar irradiance based on collected historical data from the actual research site. The collected results from the suggested algorithm indicated a significant reduction in total costs, from $17.7217 million to $13.0156 million (equivalent to 26.56% in cost reduction) for the IEEE 33-bus DS and from $18.1508 million to $13.2419 million (equivalent to 27.05% in cost reduction) for the IEEE 69-bus DS. In addition, the technical constraints are not only fully satisfied but also significantly improved, such as the weakest node voltage is increased from (0.9051 pu to 0.9523 pu) and (0.9105 pu to 0.9558 pu), the maximum THD is mitigated from (5.7491% to 4.2180%) and (5.2646% to 4.4783%), and the maximum branch current is also reduced from (0.4624 pu to 0.3217 pu) and (0.4902 pu to 0.3441 pu) for the first and second systems, respectively. In the second case, the suggested method resulted in a reduction in power loss of up to 65.51% for the first system and 69.17% for the second system, with the satisfaction of all constraints. These numerical results from both cases have proven the enormous economic-technical benefits of implementing the project of integrating WT-PVDGUs into the traditional distribution grid. Moreover, by comparing the optimal solution from MCOA with five effective implemented methods and ten recently published methods, the obtained results also confirmed that the suggested method is better and more stable than others in tackling the considered problem. The simulation results from this study have greatly contributed to affirming the multi-benefits from appropriately determining the penetration of DGUs in the power grid and beyond, also introducing a useful algorithm for solving various optimization problems.

Although this study analyzed the influence of harmonics in the distribution systems, only harmonic sources from nonlinear loads are considered. In fact, harmonics may be produced from both nonlinear loads and power conversion devices of WT-PVDGUs while connected to the grid. Therefore, in the future, harmonics from all potential sources will be considered. Besides, the high penetration of electric vehicle charging stations (EVCSs) and energy storage systems (ESSs) has seriously affected the existing grid. So, further consideration of EVCSs and ESSs, along with the presence of WT-PVDGUs, will be implemented. Besides, the integration of harmonic filters and smart inverters to enhance power quality and maximize renewables generation without technical violations is also considered in the future. Finally, novel optimization techniques such as reinforcement learning-based methods and recent data-driven approaches will be implemented to expand the problem-solving models in dynamically changing environments and large-scale historical datasets. Furthermore, hybrid optimization methods combining meta-heuristics with machine learning or mathematical programming will also be developed to improve both solution quality and computational efficiency. These are evaluated as promising directions for the next study.

**Table:** 

The list of symbols
$C_{PV}^{O\&M},\;C_{WT}^{O\&M}$ The operation & maintenance cost for PVDGU ($/MWh) and WTDGU ($/MWh)
$C_{WT}^{Cap},\;C_{PV}^{Cap}$ The capital cost for WTDGU ($/MW) and PVDGU ($/MW)
${FF}_{g,c}$, ${OF}_{g,c}$ The values of fitness and objective function from the *c*^*th*^ solution for the *g*^*th*^ group
$F_{Gbest,g}$ The best solution at the current population
$F^{Max}$, $F^{Min}$ The upper and lower limits of control variables for each solution
$F_{best,g}$, $F_{cent,g}$ The best and center solutions for the *g*^*th*^ group
$F_{g,c}^{New}$, $F_{g,c}$ The new and the old *c*^*th*^ solution in the *g*^*th*^ group
$F_{g}^{New}$ The generated new solution for the *g*^*th*^ group
$F_{r\text{1},g}$, $F_{r\text{2},g}$ The picked up solutions from the *g*^*th*^ group
$I_{DG,bh,h,y,\text{1}},\;I_{DG,bh,h,y,hr}$ *\* The fundamental frequency current and the *hr*^*th*^ order harmonic current of the *bh*^*th*^ branch at the *h*^*th*^ hour in the *y*^*th*^ year
${IHD}_{V}^{Max},\;{THD}_{V}^{Max}$ The maximum allowable limit of individual voltage harmonic distortion and total voltage harmonic distortion
${IHD}_{V,i,h,y}^{hr}$, ${THD}_{V,i,h,y}^{hr}$ Individual voltage harmonic distortion value and total voltage harmonic distortion value of the *hr*^*th*^ order harmonic at the *i*^*th*^ bus at the *h*^*th*^ hour in the *y*^*th*^ year
$I_{bh}^{Max}$ Maximum allowable current limit of the *bh*^*th*^ branch in the system
$N_{DG}$, $N_{PV},\;N_{WT}$ Total number of installed WT-PVDGUs, PVDGUs and WTDGUs
$N_{bh},\;N_{bs},\;N_{ld}$ Number of branches, buses and loads in the system
$N_{y}$ Number of years of the considered project
$P_{DG,h,y}^{grid}$, $Q_{DG,h,y}^{grid}$ The active and reactive power from main grid after connecting WT-PVDGUs at the *h*^*th*^ hour of the *y*^*th*^ year.
$P_{DG,bh,h,y}^{ls}$, $Q_{DG,bh,h,y}^{ls}$ The active and reactive power of the *bh*^*th*^ branch at the *h*^*th*^ hour of the *y*^*th*^ year after connecting WT-PVDGUs.
$P_{DG,l,h,y}^{ld}$, $Q_{DG,l,h,y}^{ld}$ The active power and the reactive power of the *l*^*th*^ load at the *h*^*th*^ hour of the *y*^*th*^ year after connecting WT-PVDGUs
$P_{PV\;}^{Min}{,\;P}_{PV}^{Max}\;$ Minimum and maximum installed active power of PVDGU
$P_{PV,p,h,y}^{ope}$, $P_{WT,w,h,y}^{ope}$ The hourly output active power of the *p*^*th*^ PVDGU and the *w*^*th*^ WTDGU at the *h*^*th*^ hour in the *y*^*th*^ year
$P_{PV,p}^{ins}$, $P_{WT,w}^{ins}$ Installed active power of the *p*^*th*^ PVDGU and the *w*^*th*^ WTDGU
${Po}_{PV}^{total}$, ${Po}_{PV}$ The expected total output power of PVDGU for a specific time period and the expected output power of PVDGU.
$P_{WT\;}^{Min}{,\;P}_{WT}^{Max}\;$ Minimum and maximum installed active power of WTDGU
${Po}_{WT}^{total}$, ${Po}_{WT}$ The expected total output power of WTDGU for a specific time period and the expected output power of WTDGU.
${Po}_{n}^{Max}$, ${Po}_{n}^{Min}$ Maximum and minimum locations of WT-PVDGU
$Q_{PV,p,h,y}^{ope},$ $Q_{WT,w,h,y}^{ope}$ The hourly output reactive power of the *p*^*th*^ PVDGU and the *w*^*th*^ WTDGU at the *h*^*th*^ hour in the *y*^*th*^ year
$R_{br}$ Resistance of the *bh*^*th*^ branch
${Si}_{n}^{Max}$, ${Si}_{n}^{Min}$ The maximum and minimum sizing of WT-PVDGU
$V_{DG,i,h,y}^{\text{1}}$, $V_{DG,i,h,y,hr}$ The voltage values of the fundamental frequency and the *hr*^*th*^ order frequency at the *i*^*th*^ bus at the *h*^*th*^ hour in the *y*^*th*^ year after connecting WT-PVDGUs
$\omega_{I}\;$, ${\Delta I}_{DG,bh,g,c}$ Penalty coefficient and penalty level of the current at the *bh*^*th*^ branch of the *c*^*th*^ solution in the *g*^*th*^ group
$\omega_{IHD},{\Delta IHD}_{V,i,g,c}$ Penalty coefficient and penalty level of the individual voltage harmonic distortion at the *i*^*th*^ bus of the *c*^*th*^ solution in the *g*^*th*^ group
$\omega_{THD}$, ${\Delta THD}_{V,i,g,c}$ Penalty coefficient and penalty level of the total voltage harmonic distortion at the *i*^*th*^ bus of the *c*^*th*^ solution in the *g*^*th*^ group
$\omega_{V}$, ${\Delta V}_{DG,i,g,c}$ Penalty coefficient and penalty level of the voltage of the *i*^*th*^ bus in the *c*^*th*^ solution for the *g*^*th*^ group
*F*_*g,c*_ The *c*^*th*^ solution in the *g*^*th*^ group
*N*_*cc*_*, N*_*mc*_*, N*_*cv*_ Close solution couple, maximum solution couple and control variables numbers
*N*_*po,*_ *N*_*gr,*_ *N*_*co*_ Population size, group number and coyote number for each group
*P*_*p*_ The output power of PVDGU
*r*_*d*_ Random number in the range [0, 1]
*W*_*r*_ Rayleigh probability density function
*ε* Predetermined threshold
Hr The maximum harmonic order number
s Stochastic wind speed (m/s)
$P_{r}$ The wind turbine’s rated power
$s_{out}$ The cut-out of wind speed of used wind turbine
$s_{in}$ The cut-in of wind speed of used wind turbine
$s_{r}$ The rated of wind speed of used wind turbine

## Supporting information

S1 File This file contains Tables S1–S9 and Figures S1–S2. Tables S1–S7 provide the data of the IEEE 33-bus and IEEE 69-bus distribution systems, average wind speed, average solar irradiance, standard deviation of irradiance, characteristics of the wind turbine and photovoltaic module, IEEE RTS-96 load profile, and the optimal solutions obtained by the applied methods. Figures S1–S2 present the hourly output power of PVDGUs and WTDGUs for the IEEE 33-bus and IEEE 69-bus distribution systems.
(DOCX)

## Abbreviation:

ABC: Artificial bee colony algorithm
ALOA: Ant lion optimization algorithm
BBO: Biogeography-based optimization
BSOA: Backtracking search optimization algorithm
CABC: Chaotic artificial bee colony algorithm
EO: Equilibrium optimizer
GRO: Garra rufa optimization
GSA: Gravitational Search Algorithm
AA: Autoadd algorithm
MOBA: Multi-objective bat algorithm
NGO: Northern Goshawk Optimization
PSO: Particle swarm optimization
PVDGU(s): Photovoltaic distributed generation unit(s)
SMA: Slime mould algorithm
GWO: Gray wolf optimization
SSA: Salp swarm algorithm
TSO: Transient search optimization
WTDGU(s): Wind turbine distributed generation unit(s)
