# Supplementary material for: Minimizing total costs for distribution power grids considering harmonics and uncertainty of wind speed and solar radiation
Source: PLoS One. 2026 Sep 11;21(9):e0355399. doi: 10.1371/journal.pone.0355399 (PMC13568534; doi:10.1371/journal.pone.0355399)
Supplement: Supplementary file 1 [file pone.0355399.s001.docx]

**Supporting Information**

**S1 Table.** Data of IEEE 33-bus distribution system

| From node | To node | Resistance  (Ω) | Reactance  (Ω) | Active power demand (kW) | Reactive power demand (kVar) |
| --- | --- | --- | --- | --- | --- |
| 1 | 2 | 0.0922 | 0.0477 | 100 | 60 |
| 2 | 3 | 0.4930 | 0.2511 | 90 | 40 |
| 3 | 4 | 0.3660 | 0.1864 | 120 | 80 |
| 4 | 5 | 0.3811 | 0.1941 | 60 | 30 |
| 5 | 6 | 0.819 | 0.7070 | 60 | 20 |
| 6 | 7 | 0.1872 | 0.6188 | 200 | 100 |
| 7 | 8 | 1.7114 | 1.2351 | 200 | 100 |
| 8 | 9 | 1.0300 | 0.7400 | 60 | 20 |
| 9 | 10 | 1.0400 | 0.7400 | 60 | 20 |
| 10 | 11 | 0.1966 | 0.0650 | 45 | 30 |
| 11 | 12 | 0.3744 | 0.1238 | 60 | 35 |
| 12 | 13 | 1.4680 | 1.1550 | 60 | 35 |
| 13 | 14 | 0.5416 | 0.7129 | 120 | 80 |
| 14 | 15 | 0.5910 | 0.5260 | 60 | 10 |
| 15 | 16 | 0.7463 | 0.5450 | 60 | 20 |
| 16 | 17 | 1.2890 | 1.7210 | 60 | 20 |
| 17 | 19 | 0.7320 | 0.5740 | 90 | 40 |
| 2 | 19 | 0.1640 | 0.1565 | 90 | 40 |
| 19 | 20 | 1.5042 | 1.3554 | 90 | 40 |
| 20 | 21 | 0.4095 | 0.4784 | 90 | 40 |
| 21 | 22 | 0.7089 | 0.9373 | 90 | 40 |
| 3 | 23 | 0.4512 | 0.3083 | 90 | 50 |
| 23 | 24 | 0.8980 | 0.7091 | 420 | 200 |
| 24 | 25 | 0.8960 | 0.7011 | 420 | 200 |
| 6 | 26 | 0.2030 | 0.1034 | 60 | 25 |
| 26 | 27 | 0.2842 | 0.1447 | 60 | 25 |
| 27 | 28 | 1.0590 | 0.9337 | 60 | 20 |
| 28 | 29 | 0.8042 | 0.7006 | 120 | 70 |
| 29 | 30 | 0.5075 | 0.2585 | 200 | 600 |
| 30 | 31 | 0.9744 | 0.9630 | 150 | 70 |
| 31 | 32 | 0.3105 | 0.3619 | 210 | 100 |

**S2 Table.** Data of IEEE 69-bus distribution system

| From node | To node | Resistance  (Ω) | Reactance  (Ω) | Active power demand at end-node (kW) | Reactive power demand at end-node (kVar) |
| --- | --- | --- | --- | --- | --- |
| 1 | 2 | 0.0005 | 0.0012 | 0 | 0 |
| 2 | 3 | 0.0005 | 0.0012 | 0 | 0 |
| 3 | 4 | 0.0015 | 0.0036 | 0 | 0 |
| 4 | 5 | 0.0251 | 0.0294 | 0 | 0 |
| 5 | 6 | 0.3660 | 0.1864 | 2.6 | 2.2 |
| 6 | 7 | 0.3811 | 0.1941 | 40.4 | 30 |
| 7 | 8 | 0.0922 | 0.0470 | 75 | 54 |
| 8 | 9 | 0.0493 | 0.0251 | 30 | 22 |
| 9 | 10 | 0.8190 | 0.2707 | 28 | 19 |
| 10 | 11 | 0.1872 | 0.0619 | 145 | 104 |
| 11 | 12 | 0.7114 | 0.2351 | 145 | 104 |
| 12 | 13 | 1.0300 | 0.3400 | 8 | 5 |
| 13 | 14 | 1.0440 | 0.3450 | 8 | 5.5 |
| 14 | 15 | 1.0580 | 0.3496 | 0 | 0 |
| 15 | 16 | 0.1966 | 0.0650 | 45.5 | 30 |
| 16 | 17 | 0.3744 | 0.1238 | 60 | 35 |
| 17 | 18 | 0.0047 | 0.0016 | 60 | 35 |
| 18 | 19 | 0.3276 | 0.1083 | 0 | 0 |
| 19 | 20 | 0.2106 | 0.0690 | 1 | 0.6 |
| 20 | 21 | 0.3416 | 0.1129 | 114 | 81 |
| 21 | 22 | 0.0140 | 0.0046 | 5 | 3.5 |
| 22 | 23 | 0.1591 | 0.0526 | 0 | 0 |
| 23 | 24 | 0.3463 | 0.1145 | 28 | 20 |
| 24 | 25 | 0.7488 | 0.2475 | 0 | 0 |
| 25 | 26 | 0.3089 | 0.1021 | 14 | 10 |
| 26 | 27 | 0.1732 | 0.0572 | 14 | 10 |
| 3 | 28 | 0.0044 | 0.0108 | 26 | 18.6 |
| 28 | 29 | 0.0640 | 0.1565 | 26 | 18.6 |
| 29 | 20 | 0.3978 | 0.1315 | 0 | 0 |
| 30 | 31 | 0.0702 | 0.0232 | 0 | 0 |
| 31 | 32 | 0.3510 | 0.1160 | 0 | 0 |
| 32 | 33 | 0.8390 | 0.2816 | 14 | 10 |
| 33 | 34 | 1.7080 | 0.5646 | 19.5 | 14 |
| 34 | 35 | 1.4740 | 0.4873 | 6 | 4 |
| 3 | 36 | 0.0044 | 0.0108 | 26 | 18.55 |
| 36 | 37 | 0.0640 | 0.11565 | 26 | 18.55 |
| 37 | 38 | 0.1053 | 0.1230 | 0 | 0 |
| 38 | 39 | 0.0304 | 0.0355 | 24 | 17 |
| 39 | 40 | 0.0018 | 0.0021 | 24 | 17 |
| 40 | 41 | 0.7283 | 0.8509 | 1.2 | 1 |
| 41 | 42 | 0.3100 | 0.3623 | 0 | 0 |
| 42 | 43 | 0.0410 | 0.0478 | 6 | 4.3 |
| 43 | 44 | 0.0092 | 0.0116 | 0 | 0 |
| 44 | 45 | 0.1089 | 0.1373 | 39.22 | 26.3 |
| 45 | 46 | 0.0009 | 0.0012 | 39.22 | 26.3 |
| 4 | 47 | 0.0034 | 0.0084 | 0 | 0 |
| 47 | 48 | 0.0851 | 0.2083 | 79 | 56.4 |
| 48 | 49 | 0.2898 | 0.7091 | 384.7 | 274.5 |
| 49 | 50 | 0.0822 | 0.2011 | 384.7 | 274.5 |
| 8 | 51 | 0.0928 | 0.0473 | 40.5 | 28.3 |
| 51 | 52 | 0.3319 | 0.1114 | 3.6 | 2.7 |
| 9 | 53 | 0.1740 | 0.0886 | 4.35 | 3.5 |
| 53 | 54 | 0.2030 | 0.1034 | 26.4 | 19 |
| 54 | 55 | 0.2842 | 0.1447 | 24 | 17.2 |
| 55 | 56 | 0.2813 | 0.1433 | 0 | 0 |
| 56 | 57 | 1.5900 | 0.5337 | 0 | 0 |
| 57 | 58 | 0.7837 | 0.2630 | 0 | 0 |
| 58 | 59 | 0.3042 | 0.1006 | 100 | 72 |
| 59 | 60 | 0.3861 | 0.1172 | 0 | 0 |
| 60 | 61 | 0.5075 | 0.2585 | 1244 | 888 |
| 61 | 62 | 0.0974 | 0.0496 | 32 | 23 |
| 62 | 63 | 0.1450 | 0.0738 | 0 | 0 |
| 63 | 64 | 0.7105 | 0.3619 | 227 | 162 |
| 64 | 65 | 1.0410 | 0.5302 | 59 | 42 |
| 11 | 66 | 0.2012 | 0.0611 | 18 | 13 |
| 66 | 67 | 0.0047 | 0.0014 | 18 | 13 |
| 12 | 68 | 0.7394 | 0.2444 | 28 | 20 |
| 68 | 69 | 0.0047 | 0.0016 | 28 | 20 |

**S3 Table.** The average wind speed (m/s) for modeling wind turbine

| Hour | Winter | Spring | Summer | Fall |
| --- | --- | --- | --- | --- |
| 1 | 7.9973 | 6.4484 | 5.4234 | 7.3186 |
| 2 | 8.0756 | 6.4214 | 5.4508 | 7.3458 |
| 3 | 8.1276 | 6.3846 | 5.4305 | 7.3088 |
| 4 | 8.1138 | 6.3784 | 5.3570 | 7.2619 |
| 5 | 8.1020 | 6.3510 | 5.2661 | 7.2487 |
| 6 | 8.1148 | 6.3277 | 5.2827 | 7.2064 |
| 7 | 8.1920 | 6.4503 | 5.5395 | 7.1714 |
| 8 | 8.2793 | 6.7445 | 5.8500 | 7.1952 |
| 9 | 8.3227 | 7.0968 | 6.0670 | 7.3096 |
| 10 | 8.3459 | 7.4097 | 6.2185 | 7.4727 |
| 11 | 8.4147 | 7.6200 | 6.3323 | 7.6262 |
| 12 | 8.4947 | 7.7403 | 6.3826 | 7.7552 |
| 13 | 8.4988 | 7.7834 | 6.3788 | 7.7809 |
| 14 | 8.4696 | 7.7399 | 6.3362 | 7.7355 |
| 15 | 8.4674 | 7.5997 | 6.2330 | 7.5948 |
| 16 | 8.4565 | 7.3419 | 6.0728 | 7.4249 |
| 17 | 8.3971 | 7.0463 | 5.8563 | 7.3334 |
| 18 | 8.3155 | 6.8460 | 5.5964 | 7.3263 |
| 19 | 8.2541 | 6.7713 | 5.4007 | 7.3273 |
| 20 | 8.2155 | 6.7518 | 5.4203 | 7.2979 |
| 21 | 8.1677 | 6.7236 | 5.4945 | 7.2856 |
| 22 | 8.118 | 6.6662 | 5.5073 | 7.3037 |
| 23 | 8.0792 | 6.5625 | 5.4832 | 7.2982 |
| 24 | 7.9831 | 6.5366 | 5.4198 | 7.2833 |

**S4 Table.** The average solar irradiance (W/m^2^) for modeling photovoltaic module

| Hour | Winter | Spring | Summer | Fall |
| --- | --- | --- | --- | --- |
| 1 | 0 | 0 | 0 | 0 |
| 2 | 0 | 0 | 0 | 0 |
| 3 | 0 | 0 | 0 | 0 |
| 4 | 0 | 6.0406 | 24.395 | 0 |
| 5 | 0 | 43.845 | 99.607 | 0.23363 |
| 6 | 0 | 124.39 | 222.59 | 15.225 |
| 7 | 0.6451 | 247.58 | 363.69 | 70.007 |
| 8 | 17.336 | 387.02 | 501.4 | 162.06 |
| 9 | 77.958 | 512.11 | 621.79 | 266.13 |
| 10 | 157.42 | 609.15 | 714.26 | 349.33 |
| 11 | 212.73 | 668.68 | 771.00 | 397.4 |
| 12 | 231.17 | 679.47 | 780.98 | 402.56 |
| 13 | 211.11 | 654.03 | 757.57 | 369.04 |
| 14 | 154.98 | 587.02 | 695.22 | 297.38 |
| 15 | 79.025 | 484.3 | 598.12 | 199.3 |
| 16 | 24.419 | 355.39 | 475.33 | 103.71 |
| 17 | 1.7147 | 215.3 | 337.93 | 37.71 |
| 18 | 0 | 95.227 | 200.20 | 5.619 |
| 19 | 0 | 26.212 | 83.711 | 0 |
| 20 | 0 | 1.6934 | 15.58 | 0 |
| 21 | 0 | 0 | 0 | 0 |
| 22 | 0 | 0 | 0 | 0 |
| 23 | 0 | 0 | 0 | 0 |
| 24 | 0 | 0 | 0 | 0 |

**S5 Table.** The standard deviation of irradiance (W/m^2^) for modeling photovoltaic module

| Hour | Winter | Spring | Summer | Fall |
| --- | --- | --- | --- | --- |
| 1 | 0 | 0 | 0 | 0 |
| 2 | 0 | 0 | 0 | 0 |
| 3 | 0 | 0 | 0 | 0 |
| 4 | 0 | 11.041 | 21.838 | 0 |
| 5 | 0 | 51.833 | 52.32 | 1.107 |
| 6 | 0 | 102.48 | 65.95 | 26.61 |
| 7 | 2.45 | 133.13 | 69.27 | 75.918 |
| 8 | 31.408 | 137.72 | 69.059 | 117.67 |
| 9 | 64.022 | 134.7 | 67.212 | 134.94 |
| 10 | 77.487 | 130.00 | 65.512 | 143.28 |
| 11 | 85.663 | 125.00 | 64.152 | 148.43 |
| 12 | 89.616 | 120.09 | 63.618 | 149.9 |
| 13 | 92.467 | 116.53 | 63.071 | 151.67 |
| 14 | 91.272 | 114.60 | 63.496 | 150.23 |
| 15 | 78.648 | 114.03 | 64.824 | 141.38 |
| 16 | 39.655 | 113.24 | 66.354 | 108.88 |
| 17 | 4.9772 | 107.99 | 66.202 | 57.585 |
| 18 | 0 | 81.244 | 61.878 | 13.773 |
| 19 | 0 | 34.904 | 45.747 | 0 |
| 20 | 0 | 4.0223 | 13.606 | 0 |
| 21 | 0 | 0 | 0 | 0 |
| 22 | 0 | 0 | 0 | 0 |
| 23 | 0 | 0 | 0 | 0 |
| 24 | 0 | 0 | 0 | 0 |

**S6 Table.** The characteristics of the wind turbine

| Rated power | Cut-in wind speed | Rated wind speed | Cut-out wind speed |
| --- | --- | --- | --- |
| 100 kW | 3 m/s | 10 m/s | 20 m/s |

**S7 Table.** The characteristics of the photovoltaic module

| Item | Parameters |
| --- | --- |
| Peak watt (W) | 75 |
| Open circuit volt (V) | 21.98 |
| Short circuit current (A) | 5.32 |
| Voltage at maximum power (V) | 17.32 |
| Current at maximum power (A) | 4.76 |
| Volt temperature coefficient (mV/^o^C) | 14.4 |
| Current temperature coefficient (mA/^o^C) | 1.22 |
| Nominal cell operating temperature (^o^C) | 43.00 |

**S8 Table.** Load profile of IEEE RTS-96

| Hour | Winter | Spring | Summer | Fall |
| --- | --- | --- | --- | --- |
| 1 | 0.4757 | 0.3969 | 0.64 | 0.3717 |
| 2 | 0.4473 | 0.3906 | 0.6 | 0.3658 |
| 3 | 0.426 | 0.378 | 0.58 | 0.354 |
| 4 | 0.4189 | 0.3654 | 0.56 | 0.3422 |
| 5 | 0.4189 | 0.3717 | 0.56 | 0.3481 |
| 6 | 0.426 | 0.4095 | 0.58 | 0.3835 |
| 7 | 0.5254 | 0.4536 | 0.64 | 0.4248 |
| 8 | 0.6106 | 0.5355 | 0.76 | 0.5015 |
| 9 | 0.6745 | 0.5985 | 0.87 | 0.5605 |
| 10 | 0.6816 | 0.6237 | 0.95 | 0.5841 |
| 11 | 0.6816 | 0.63 | 0.99 | 0.59 |
| 12 | 0.6745 | 0.6237 | 1 | 0.5841 |
| 13 | 0.6745 | 0.5859 | 0.99 | 0.5487 |
| 14 | 0.6745 | 0.5796 | 1 | 0.5428 |
| 15 | 0.6603 | 0.567 | 1 | 0.531 |
| 16 | 0.6674 | 0.5544 | 0.97 | 0.5192 |
| 17 | 0.7029 | 0.567 | 0.96 | 0.531 |
| 18 | 0.71 | 0.5796 | 0.96 | 0.5428 |
| 19 | 0.71 | 0.6048 | 0.93 | 0.5664 |
| 20 | 0.6816 | 0.6174 | 0.92 | 0.5782 |
| 21 | 0.6461 | 0.6048 | 0.92 | 0.5664 |
| 22 | 0.5893 | 0.567 | 0.93 | 0.531 |
| 23 | 0.5183 | 0.504 | 0.87 | 0.472 |
| 24 | 0.4473 | 0.441 | 0.72 | 0.413 |

**S9 Table.** Optimal solutions obtained by applied methods for two systems

| Method | IEEE 33- bus distribution system | | IEEE 69- bus distribution system | |
| --- | --- | --- | --- | --- |
|  | PVDGUs | WTDGUs | PVDGUs | WTDGUs |
| EO | Bus 09 – 2318 modules  Bus 06 – 11003 modules  Bus 15 – 2489 modules | Bus 31 – 2 turbines  Bus 12 – 15 turbines  Bus 33 – 06 turbines | Bus 49 – 7150 modules  Bus 54 – 6351 modules  Bus 02 – 2568 modules | Bus 64 – 02 turbines  Bus 61 – 14 turbines  Bus 63 – 08 turbines |
|  | Total: 1,185,750 (W) | Total: 2,300 (kW) | Total: 1,205,175 (W) | Total: 2,400 (kW) |
| TSO | Bus 05 – 3184 modules  Bus 02 – 7908 modules  Bus 11 – 2100 modules | Bus 31 – 02 turbines  Bus 12 – 15 turbines  Bus 33 – 06 turbines | Bus 60 – 5528 modules  Bus 56 – 4433 modules  Bus 23 – 4534 modules | Bus 69 – 02 turbines  Bus 62 – 14 turbines  Bus 64 – 08 turbines |
|  | Total: 989,400 (W) | Total: 2,300 (kW) | Total: 1,087,125 (W) | Total: 2,400 (kW) |
| SMA | Bus 30 – 1000 modules  Bus 33 – 12843 modules  Bus 14 – 1923 modules | Bus 32 – 03 turbines  Bus 29 – 06 turbines  Bus 14 – 14 turbines | Bus 54 – 5744 modules  Bus 50 – 5483 modules  Bus 03 – 4414 modules | Bus 63 – 02 turbine  Bus 62 – 14 turbines  Bus 64 – 08 turbines |
|  | Total: 1,182,450 (W) | Total: 2,300 (kW) | Total: 1,173,075 (W) | Total: 2,400 (kW) |
| NGO | Bus 02 – 3163 modules  Bus 05 – 9891 modules  Bus 11 – 1000 modules | Bus 31 – 02 turbines  Bus 10 – 15 turbines  Bus 33 – 06 turbines | Bus 55 – 6069 modules  Bus 54 – 6135 modules  Bus 20 – 2795 modules | Bus 63 – 02 turbines  Bus 62 – 14 turbines  Bus 64 – 08 turbines |
|  | Total: 1,054,050 (W) | Total: 2,300 (kW) | Total: 1,124,925 (W) | Total: 2,400 (kW) |
| COA | Bus 03 – 1016 modules  Bus 26 – 6748 modules  Bus 27 – 8181 modules | Bus 31 – 04 turbines  Bus 29 – 10 turbines  Bus 14 – 09 turbines | Bus 60 – 5744 modules  Bus 67 – 4682 modules  Bus 11 – 5624 modules | Bus 53 – 02 turbines  Bus 60 – 13 turbines  Bus 63 – 09 turbines |
|  | Total: 1,195,875 (W) | Total: 2,300 (kW) | Total: 1,203,750 (W) | Total: 2,400 (kW) |
| MCOA | Bus 06 – 13959 modules  Bus 04 – 1000 modules  Bus 31 – 1233 modules | Bus 15 – 09 turbines  Bus 28 – 02 turbines  Bus 30 – 12 turbines | Bus 17 – 5746 modules  Bus 64 – 4683 modules  Bus 13 – 5645 modules | Bus 53 – 02 turbines  Bus 62 – 12 turbines  Bus 61 – 10 turbines |
|  | Total: 1,214,400 (W) | Total: 2,300 (kW) | Total: 1,205,550 (W) | Total: 2,400 (kW) |

**S1 Figure.** The hourly output power of PVDGUs and WTDGUs in IEEE 33-bus DS

**S2 Figure.** The hourly output power of PVDGUs and WTDGUs in IEEE 69-bus DS
